# Deucravacitinib, a tyrosine kinase 2 pseudokinase inhibitor, protects human EndoC-βH1 β-cells against proinflammatory insults

**DOI:** 10.3389/fimmu.2023.1263926

**Published:** 2023-10-03

**Authors:** Reinaldo S. Dos Santos, Daniel Guzman-Llorens, Atenea A. Perez-Serna, Angel Nadal, Laura Marroqui

**Affiliations:** ^1^ Instituto de Investigación, Desarrollo e Innovación en Biotecnología Sanitaria de Elche (IDiBE), Universidad Miguel Hernández de Elche, Alicante, Spain; ^2^ CIBER de Diabetes y Enfermedades Metabólicas Asociadas, Instituto de Salud Carlos III, Madrid, Spain

**Keywords:** apoptosis, deucravacitinib, inflammation, pancreatic β-cells, TYK2, type 1 diabetes, type I interferons

## Abstract

**Introduction:**

Type 1 diabetes is characterized by pancreatic islet inflammation and autoimmune-driven pancreatic β-cell destruction. Interferon-α (IFNα) is a key player in early human type 1 diabetes pathogenesis. IFNα activates the tyrosine kinase 2 (TYK2)-signal transducer and activator of transcription (STAT) pathway, leading to inflammation, HLA class I overexpression, endoplasmic reticulum (ER) stress, and β-cell apoptosis (in synergy with IL-1β). As TYK2 inhibition has raised as a potential therapeutic target for the prevention or treatment of type 1 diabetes, we investigated whether the selective TYK2 inhibitor deucravacitinib could protect β-cells from the effects of IFNα and other proinflammatory cytokines (i.e., IFNγ and IL-1β).

**Methods:**

All experiments were performed in the human EndoC-βH1 β-cell line. HLA class I expression, inflammation, and ER stress were evaluated by real-time PCR, immunoblotting, and/or immunofluorescence. Apoptosis was assessed by the DNA-binding dyes Hoechst 33342 and propidium iodide or caspase 3/7 activity. The promoter activity was assessed by luciferase assay.

**Results:**

Deucravacitinib prevented IFNα effects, such as STAT1 and STAT2 activation and MHC class I hyperexpression, in a dose-dependent manner without affecting β-cell survival and function. A comparison between deucravacitinib and two Janus kinase inhibitors, ruxolitinib and baricitinib, showed that deucravacitinib blocked IFNα- but not IFNγ-induced signaling pathway. Deucravacitinib protected β-cells from the effects of two different combinations of cytokines: IFNα + IL-1β and IFNγ + IL-1β. Moreover, this TYK2 inhibitor could partially reduce apoptosis and inflammation in cells pre-treated with IFNα + IL-1β or IFNγ + IL-1β.

**Discussion:**

Our findings suggest that, by protecting β-cells against the deleterious effects of proinflammatory cytokines without affecting β-cell function and survival, deucravacitinib could be repurposed for the prevention or treatment of early type 1 diabetes.

## 1 Introduction

Type 1 diabetes is characterized by pancreatic islet inflammation and specific destruction of pancreatic β-cells by an autoimmune assault, which develops in the context of an inadequate “dialogue” between β-cells and the invading immune cells (1, 2).

A growing body of evidence places type I interferons (IFNs) as key players in the early stages of human type 1 diabetes pathogenesis (3). IFNα was found in islets from type 1 diabetes patients (4–6), and laser-captured islets from living donors with recent-onset type 1 diabetes showed increased expression of IFN-stimulated genes (ISGs) (7). In genetically susceptible children, an IFN signature was temporarily amplified preceding the development of autoantibodies and throughout the progress of type 1 diabetes (8, 9). Recently, three type I IFN response markers, namely human MX Dynamin Like GTPase 1 (MX1), double-stranded RNA sensor protein kinase R, and HLA class I, were found to be expressed in a significantly higher percentage of insulin-containing islets from autoantibody-positive and/or recent-onset type 1 diabetes donors (10). In human β-cells, IFNα induced inflammation, endoplasmic reticulum (ER) stress as well as a long-lasting overexpression of HLA class I via activation of the tyrosine kinase 2 (TYK2)-signal transducer and activator of transcription (STAT) pathway. Moreover, IFNα induced apoptosis in the presence of IL-1β (11–14).

Targeting the type I IFN signaling pathway has been proposed as a potential adjuvant therapy to treat at-risk individuals or patients still in the very early stages of the disease (3, 15). Among some of the strategies that have been suggested, inhibitors of Janus kinase (JAK) proteins (JAK1-3 and TYK2) show great promise. Treatment with AZD1480 (a JAK1/JAK2 inhibitor) and ABT 317 (a JAK1-selective inhibitor) protected non-obese diabetic mice against autoimmune diabetes and reversed diabetes in newly diagnosed non-obese diabetic mice (16, 17). In human β-cells, clinically used JAK inhibitors, namely ruxolitinib, cerdulatinib, and baricitinib, prevented MHC class I overexpression, ER stress, chemokine production, and apoptosis (13, 14).

Lately, attention has focused on *TYK2*, a candidate gene for type 1 diabetes whose genetic variants that decrease TYK2 activity are associated with protection against the disease (18–20). TYK2 is crucial for cell development and IFNα-mediated responses in human β-cells (11, 21, 22). Partial TYK2 knockdown protected human β-cells against apoptosis and inflammation induced by polyinosinic-polycitidilic acid, a mimic of double-stranded RNA produced during viral infection (21). In mature stem cell-islets, TYK2 knockout or pharmacologic inhibition decreased T-cell-mediated cytotoxicity by preventing IFNα-induced antigen processing and presentation, including MHC class I expression (22). As these findings place TYK2 as a critical regulator of the type I IFN signaling pathway in β-cells, selective TYK2 inhibition has emerged as a drug target to treat type 1 diabetes. Recently, two novel small molecule inhibitors binding to the TYK2 pseudokinase domain protected human β-cells against the deleterious effects of IFNα without compromising β-cell function and susceptibility to potentially diabetogenic viruses (23).

Deucravacitinib, a small molecule that selectively targets the TYK2 pseudokinase domain, has shown great therapeutic potential for immune-mediated diseases, such as lupus nephritis and systemic lupus erythematosus (24, 25). In fact, deucravacitinib has been recently approved for treatment of plaque psoriasis (26). However, no preclinical studies have deeply explored the possible use of deucravacitinib in the context of type 1 diabetes. Notably, Chandra et al. recently used deucravacitinib to validate their CRISPR-Cas9-generated *TYK2* knockout in human induced pluripotent stem cells, but did not provide further characterisation of its effects on β-cells (22).

In this study, we report the effects of deucravacitinib on the human insulin-producing EndoC-βH1 cells, including its ability to prevent IFNα-triggered signaling pathway and damaging effects on β-cells.

## 2 Materials and methods

### 2.1 Culture of EndoC-βH1 cells

The human EndoC-βH1 β-cell line [research resource identifier (RRID): CVCL_L909, Univercell-Biosolutions, France] was cultured in Matrigel/fibronectin-coated plates as previously described (27). Cells were cultured in DMEM containing 5.6 mmol/L glucose, 10 mmol/L nicotinamide, 5.5 μg/mL transferrin, 50 μmol/L 2-mercaptoethanol, 6.7 ng/mL selenite, 2% BSA fatty acid free, 100 U/mL penicillin, and 100 μg/mL streptomycin. We confirmed that cells were mycoplasma-free using the MycoAlert Mycoplasma Detection Kit (Lonza, Basel, Switzerland).

### 2.2 Cell treatments

Proinflammatory cytokine concentrations were selected according to previously established experiments in human β-cells (11, 28): recombinant human IFNα (PeproTech Inc., Rocky Hill, NJ) at 1000 U/mL; recombinant human IFNγ (PeproTech Inc., Rocky Hill, NJ) at 1000 U/mL; and recombinant human IL-1β (R&D Systems, Abingdon, UK) at 50 U/mL. Ruxolitinib, baricitinib, or deucravacitinib (Selleckchem, Planegg, Germany) were prepared in DMSO (used as vehicle) and cells were treated as indicated in the figures. Ruxolitinib and baricitinib concentrations were selected based on previous dose-response experiments (unpublished data). For treatments involving cytokines, 2% FBS was added to the culture medium.

### 2.3 Cell viability assessment

The percentage of apoptosis was measured by fluorescence microscopy upon staining with the DNA-binding dyes Hoechst 33342 and propidium iodide (Sigma-Aldrich, Saint Louis, MO, USA) as described (29). At least 600 cells were counted for each experimental condition. Viability was assessed by two independent researchers, one of whom was unaware of sample identity, with >90% agreement between results.

### 2.4 Caspase 3/7 activity

Caspase 3/7 activity was determined using the Caspase-Glo^®^ 3/7 assay (Promega, Madison, WI, USA) following the manufacturer’s instructions. Briefly, upon incubation in 100 µL culture medium, cells were incubated with 100 µL Caspase-Glo^®^ 3/7 reagent at room temperature for 1 h before recording luminescence with a POLASTAR plate reader (BMG Labtech, Ortenberg, Germany).

### 2.5 C-X-C motif chemokine ligand 10 measurements

The release of C-X-C motif chemokine ligand 10 (CXCL10) to the culture medium was detected using Human ProcartaPlex immunoassays (Invitrogen, Vienna, Austria) following the manufacturer’s recommendations. Reactions were read with a MagPix system (Luminex, Austin, TX, USA).

### 2.6 Luciferase reporter assays

Cells were transfected using Lipofectamine 2000 (Invitrogen) with pRL-CMV encoding *Renilla* luciferase (Promega) and luciferase reporter constructs for either gamma-interferon activation site (GAS) (Panomics, Fremont, CA, USA) or IFN-stimulated regulatory element (ISRE) (kindly provided by Dr Izortze Santin, University of the Basque Country, Spain). After recovery, cells were treated with either IFNα for 2 h or IFNγ for 24 h (30). Luciferase activity was measured in a POLASTAR plate reader (BMG Labtech) using the Dual-Luciferase Reporter Assay System (Promega) and corrected for the luciferase activity of the internal control plasmid, i.e., pRL-CMV.

### 2.7 Real-time PCR

Poly(A)^+^ mRNA was extracted using Dynabeads mRNA DIRECT kit (Invitrogen) and cDNA synthesis was performed using the High-Capacity cDNA Reverse Transcription Kit (Applied Biosystems). Real-time PCR was performed on the CFX96 Real Time System (Bio-Rad) as described (31) and the housekeeping gene β-actin was used to correct expression values. Of note, β-actin expression was not altered by the experimental conditions used herein. All primers used here are listed in **Supplementary Table 1**.

### 2.8 Immunoblotting and immunofluorescence analyses

Western blotting analysis was performed as described (32). Briefly, cells were washed with cold PBS and lysed in Laemmli buffer. Immunoblotting was performed using antibodies against phospho-STAT1 (P-STAT1), phospho-STAT2 (P-STAT2), STAT1, STAT2 (all at 1:1000 dilution), and α-tubulin (1:5000). Peroxidase-conjugated antibodies (1:5000) were used as secondary antibodies. SuperSignal West Femto chemiluminescent substrate (Thermo Scientific, Rockford, IL, USA) and ChemiDoc XRS+ (Bio-Rad Laboratories, Hercules, CA, USA) were used to detect bands.

Immunofluorescence was carried out as described (21, 33). First, cells were washed with cold PBS and fixed with 4% paraformaldehyde. Afterwards, cells were permeabilised and incubated with the mouse anti-MHC Class I (W6/32) antibody (1:1000). The Alexa Fluor 568 polyclonal goat anti-mouse IgG was used as secondary antibody and Hoechst 33342 for counterstaining. Coverslips were mounted with fluorescent mounting medium (Dako, Carpintera, CA, USA) and images were taken on a Zeiss LSM900 microscope with Airyscan 2 (Zeiss-Vision, Munich, Germany) and a x40 objective. Quantification was performed using ZEN (version 3.3; Zeiss-Vision) and open-source FIJI (version 2.0; https://fiji.sc) softwares.

All antibodies used here are listed in **Supplementary Table 2**.

All the original, uncropped images representing immunoblots and microscopic photos are provided in the Supplementary Material.

### 2.9 Glucose-stimulated insulin secretion

After preincubation in modified Krebs-Ringer for 1 h, cells were sequentially stimulated with low (0 mmol/L) and high glucose (20 mmol/L) for 1 h (each stimulation) as previously described (34). Insulin secreted and insulin content from lysed cells were measured using a human insulin ELISA kit (Mercodia, Uppsala, Sweden) following the manufacturer’s instructions. The amount of secreted insulin as *% of total insulin* was calculated as previously described (35) and data were normalized to insulin secretion at 20 mmol/L glucose in vehicle-treated cells without IFNα (considered as 100%). See Supplementary Material for further details.

### 2.10 Statistical analyses

The GraphPad Prism 7.0 software (GraphPad Software, La Jolla, CA, USA) was used for statistical analyses. Data are shown as mean ± SEM of independent experiments (*i.e.* considering EndoC-βH1 cells from different passages as *n = 1*). The statistical significance of differences between groups was evaluated using one-way ANOVA followed by Dunnett’s test or two-way ANOVA followed by Sidak’s test or Dunnett’s test, as appropriate. Differences were considered statistically significant when p ≤ 0.05.

## 3 Results

### 3.1 Deucravacitinib prevented IFNα effects without affecting β-cell survival and function

**Figure 1:**
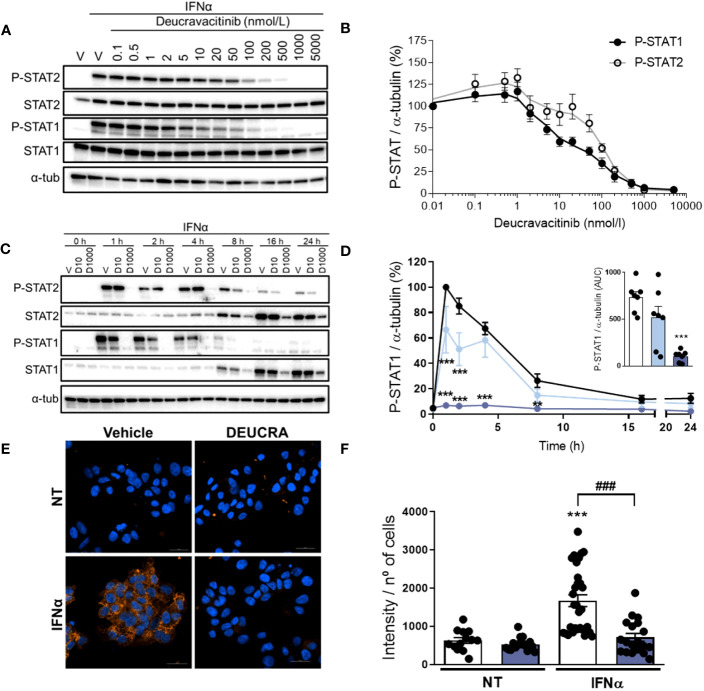
Deucravacitinib inhibits IFNα-mediated STAT phosphorylation and MHC class I overexpression. **(A, B)**: EndoC-βH1 cells were treated with vehicle (V) or pre-treated with the indicated deucravacitinib concentrations for 1 h. Afterwards, cells were left non-treated or treated with IFNα (1000 U/mL) in the absence or presence of deucravacitinib for 1 h. Representative immunoblots of P-STAT2, STAT2, P-STAT1, STAT1, and α-tubulin **(A)**, and quantification of P-STAT1 (black circles) and P-STAT2 (white circles) **(B)**. Values were normalized to α-tubulin, and then to the value of IFNα alone of each experiment (considered as 100%) (*n* = 4-6 independent experiments). **(C–F)**: EndoC-βH1 cells were treated with vehicle (V or Veh, black circles) or pre-treated with deucravacitinib (10 [D10, soft blue circles] and 1000 nmol/L [D1000, dark blue circles]) for 1 h. Afterwards, cells were left non-treated or treated with IFNα (1000 U/mL) in the absence or presence of deucravacitinib for 1–24 h **(C, D)** or 24 h **(E, F)**. **(C, D)**: Representative immunoblots of P-STAT2, STAT2, P-STAT1, STAT1, and α-tubulin **(C)**, and quantification of P-STAT1 **(D)**. The inset in **(D)** is the area under curve (AUC) of P-STAT1. Values were normalized to α-tubulin, and then to the highest value of each experiment (considered as 1) (*n* = 3-7 independent experiments). **(E, F)**: Immunocytochemistry analysis of MHC class I (red) and Hoechst 33342 (blue) upon exposure to IFNα in the absence (white bars) or presence of 1000 nmol/L deucravacitinib (dark blue bars) for 24 h. Representative images **(E)** and quantification **(F)** of MHC class I are shown (*n* = 13-30 images/coverslip from 3 different independent experiments). Data are mean ± SEM. *D*: **p ≤ 0.01, ***p ≤ 0.001 vs. Vehicle + IFNα (two-way ANOVA plus Dunnett’s test). F: vs. the respective non-treated (NT) (two-way ANOVA plus Sidak’s test); ^###^p ≤ 0.001, as indicated by bars (two-way ANOVA plus Dunnett’s test).

IFNα-mediated TYK2 activation leads to STAT1 and STAT2 phosphorylation, which will eventually upregulate several ISGs, including *HLA-ABC*, *CXCL10*, and *MX1* (**Supplementary Figure 1A**). Pre-treatment with deucravacitinib inhibited IFNα-induced STAT1 and STAT2 phosphorylation in a dose-dependent manner, where deucravacitinib showed greater potency against IFNα-stimulated STAT1 phosphorylation (**Figures 1A, B**). We then selected two doses, 10 and 1000 nmol/L, for the follow-up experiments. Next, we examined how deucravacitinib affects the kinetics of IFNα-induced STAT activation. IFNα increased P-STAT1 and P-STAT2 levels, with a maximum effect at 1-4 h post-treatment and a return to baseline by 24 h (**Figures 1C, D**; **Supplementary Figure 1B**). Although STAT1 and STAT2 protein levels were already upregulated by 8 h, STAT2 expression reached peak level at 16 h, while STAT1 expression was still increasing by 24 h (**Supplementary Figures 1C, D**). Exposure to 1000 nmol/L deucravacitinib abrogated the IFNα-stimulated STAT1 and STAT2 phosphorylation and protein expression, whereas 10 nmol/L deucravacitinib had only a minor effect (**Figures 1C, D** and **Supplementary Figures 1B–D**). Furthermore, IFNα-induced MHC class I protein overexpression was blocked by 1000 nmol/L deucravacitinib (**Figures 1E, F**). Finally, deucravacitinib did not affect β-cell viability nor changed glucose-stimulated insulin secretion and insulin content in the absence or presence of IFNα (**Supplementary Figures 1E–G**).

### 3.2 IFNα, but not IFNγ signaling pathway was blocked by deucravacitinib

**Figure 2:**
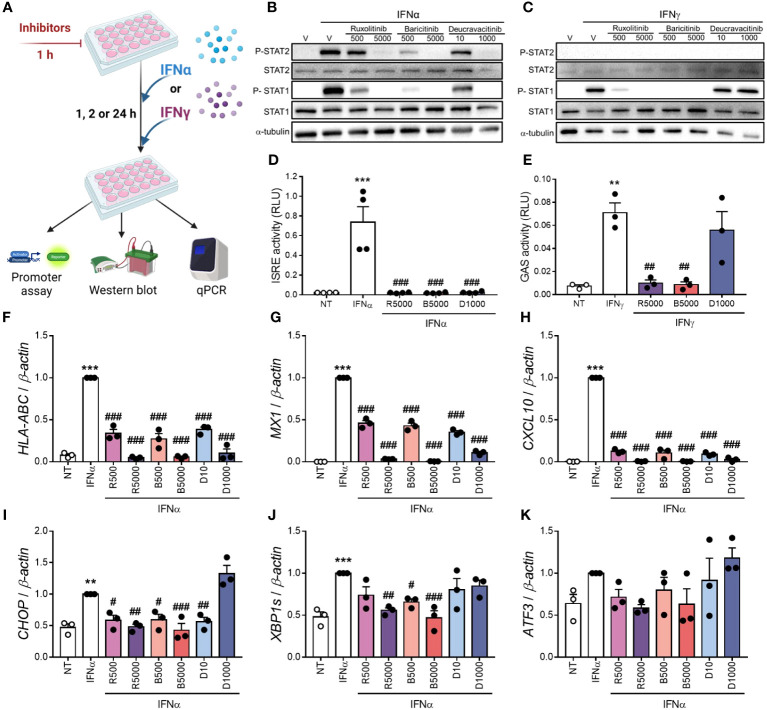
Deucravacitinib blocks IFNα- but not IFNγ-induced pathway. **(A)**: Experimental design of the pre-treatment with deucravacitinib and subsequent exposure to IFNα or IFNγ for 1, 2 or 24 h. EndoC-βH1 cells were treated with vehicle (V, white bars) or pre-treated with ruxolitinib (500 and 5000 nmol/L; R500 and R5000), baricitinib (500 and 5000 nmol/L; B500 and B5000), or deucravacitinib (10 and 1000 nmol/L; D10 and D1000) for 1 h. **(B, C)**: After the pre-treatment, cells were left non-treated (NT, white circles) or treated with either IFNα (1000 U/mL) **(B)** or IFNγ (1000 U/mL) **(C)** in the absence or presence of each inhibitor for 1 h. Representative immunoblots of P-STAT2, STAT2, P-STAT1, STAT1, and α-tubulin (*n* = 4-6 independent experiments). **(D, E)**: EndoC-βH1 cells were transfected with a pRL-CMV plasmid (used as internal control) plus either ISRE **(D)** or GAS **(E)** promoter reporter constructs. After 48 h of recovery, cells were pre-treated as described in **(A)** After the pre-treatment, cells were left non-treated (NT, white circles) or treated with either IFNα (1000 U/mL) for 2 h **(D)** or IFNγ (1000 U/mL) for 24 h **(E)** in the absence or presence of each inhibitor. Relative luciferase units (RLU) were measured by a luminescent assay (*n* = 3-4 independent experiments). **(F**–**K)**: EndoC-βH1 cells were pre-treated as described in **(A)** After the pre-treatment, cells were left non-treated (NT) or treated with IFNα (1000 U/mL) in the absence or presence of each inhibitor for 24 h. mRNA expression of *HLA-ABC* **(F)**, *MX1* **(G)**, *CXCL10* **(H)**, *CHOP* **(I)**, *XBP1s* **(J)**, and *ATF3* **(K)** was analyzed by real-time PCR, normalized to β-actin and then to the value of IFNα alone of each experiment (considered as 1) (*n* = 3 independent experiments). Data are mean ± SEM. **p ≤ 0.01, ***p ≤ 0.01 vs. the respective non-treated (NT) (one-way ANOVA plus Dunnett’s test). ^#^p ≤ 0.05, ^##^p ≤ 0.01, ^###^p ≤ 0.001 vs. IFNα **(D, F–K)** or IFNγ **(E)** (one-way ANOVA plus Dunnett’s test).

We compared deucravacitinib with ruxolitinib and baricitinib, two JAK1/JAK2 inhibitors previously tested in β-cells (13, 14). First, we measured the levels of P-STAT1 and P-STAT2 upon stimulation with IFNα or IFNγ (**Figures 2A–C**; **Supplementary Figure 2**). Ruxolitinib, baricitinib, and deucravacitinib prevented IFNα-stimulated increase in P-STAT1 and P-STAT2 levels (**Figure 2B**; **Supplementary Figures 2A, B**). Nevertheless, deucravacitinib did not change IFNγ-induced STAT1 phosphorylation, whereas ruxolitinib and baricitinib blocked it (**Figure 2C**; **Supplementary Figure 2C**). We next assessed ISRE and GAS reporter activities upon stimulation with IFNα or IFNγ (**Figures 2D, E**). While all three inhibitors abrogated IFNα-stimulated ISRE reporter activity (**Figure 2D**), IFNγ-induced GAS activation was barely affected by deucravacitinib (**Figure 2E**). As TYK2 is not involved in the IFNγ-triggered signaling pathway, the lack of deucravacitinib effect in IFNγ-treated cells is expected.

### 3.3 Deucravacitinib blocked IFNα-induced upregulation of ISGs, but not ER stress markers

Assessement of the expression of some ISGs and ER stress markers showed that all three inhibitors prevented IFNα-induced upregulation of *HLA-ABC*, *CXCL10*, and *MX1* in a dose-dependent manner (**Figures 2F–K**). Although ruxolitinib and baricitinib inhibited the mRNA expression of the ER stress markers C/EBP homologous protein (*CHOP*) and spliced isoform of XBP1 X-box binding protein 1 (*XBP1s*), only 10 nmol/L deucravacitinib reduced *CHOP* expression (**Figures 2I, J**). None of these inhibitors changed the expression of activating transcription factor 3 (*ATF3*) (**Figure 2K**).

### 3.4 Deucravacitinib prevented cytokine-induced effects in β-cells

**Figure 3:**
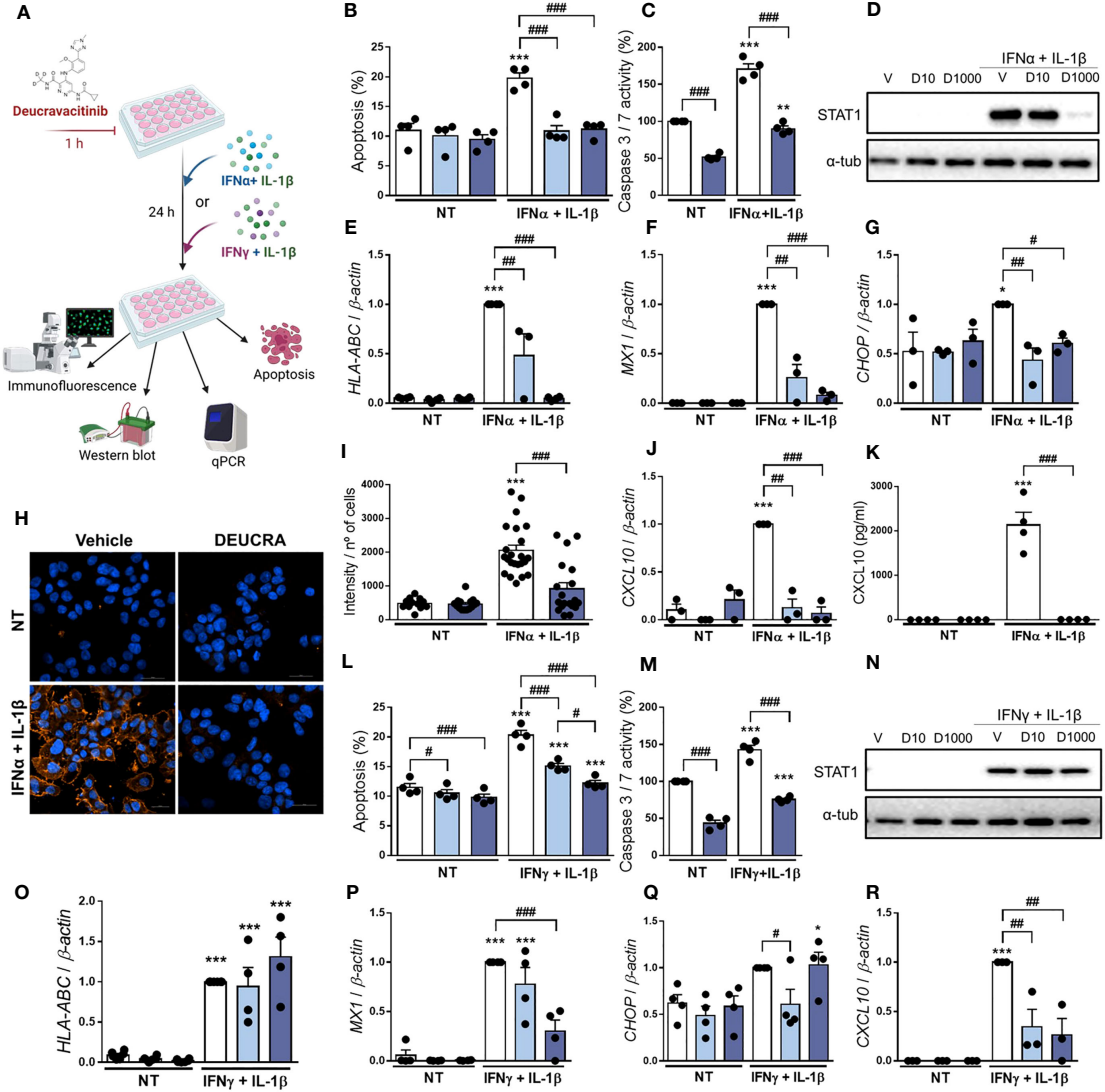
Pre-treatment with deucravacitinib prevents IFNα + IL-1β or IFNγ + IL-1β effects. **(A)**: Experimental design of the pre-treatment with deucravacitinib and subsequent exposure to cytokines for 24 h. EndoC-βH1 cells were treated with vehicle (V, white bars) or pre-treated with deucravacitinib (10 [D10, soft blue bars] and 1000 nmol/L [D1000, dark blue bars]) for 1 h. Afterwards, cells were left non-treated (NT) or treated with IFNα + IL-1β (1000 U/mL + 50 U/mL, respectively) **(B–K)** or IFNγ + IL-1β (1000 U/mL + 50 U/mL, respectively) **(L–R)** in the absence or presence of deucravacitinib for 24 h. **(B, L)**: Apoptosis was evaluated using Hoechst 33342/propidium iodide staining (*n* = 4 independent experiments). **(C, M)**: Caspase 3/7 activity was measured by a luminescent assay. Results are expressed as % vehicle-treated cells in the absence of cytokines (NT) (*n* = 4 independent experiments). **(D, N)**: Representative immunoblots of P-STAT1, STAT1, and α-tubulin (*n* = 4 independent experiments). **(E–G, J, O-R)**: mRNA expression of *HLA-ABC* **(E, O)**, *MX1* **(F, P)**, *CHOP* **(G, Q)**, and *CXCL10* **(J, R)** was analyzed by real-time PCR, normalized to β-actin and then to the value of Vehicle treated with IFNα + IL-1β **(E–G, J)** or IFNγ + IL-1β **(O-R)** (considered as 1) (*n* = 3-4 independent experiments). **(H, I)**: Immunocytochemistry analysis of MHC class I (red) and Hoechst 33342 (blue) upon exposure to IFNα + IL-1β in the absence (white bars) or presence of deucravacitinib (dark blue bars) for 24 h. Representative images **(H)** and quantification **(I)** of MHC class I are shown (12–23 images/coverslip from 3 different independent experiments). **(K)**: CXCL10 secreted to the medium was determined by ELISA (*n* = 4 independent experiments). Data are mean ± SEM. *p ≤ 0.05, **p ≤ 0.01, ***p ≤ 0.001 vs. the respective non-treated (NT) (two-way ANOVA plus Sidak’s test). ^#^p ≤ 0.05, ^##^p ≤ 0.01, ^###^p ≤ 0.001, as indicated by bars (two-way ANOVA plus Dunnett’s test).

Previous studies showed that a combination of IFNα + IL-1β, two cytokines that might be present in the islet milieu at early stages of insulitis, induces β-cell apoptosis, inflammation, and ER stress (11, 14, 23). Thus, we investigated whether deucravacitinib protects β-cells after IFNα + IL-1β exposure (**Figure 3A**). We observed that deucravacitinib completely prevented IFNα + IL-1β-induced apoptosis (**Figures 3B, C**). Moreover, deucravacitinib-treated cells showed reduced levels of P-STAT1 and STAT1 (**Figure 3D**; **Supplementary Figures 3A, B**) as well as *HLA-ABC*, *MX1*, *CHOP*, and *CXCL10* mRNA expression (**Figures 3E–G, J**). MHC class I protein expression and CXCL10 secretion were also decreased by TYK2 inhibition (**Figures 3H, I, K**).

We next evaluated whether deucravacitinib protects against cytokines that, as compared with IFNα, probably appear later in the progression of islet inflammation: IFNγ and IL-1β (36). After treatment for 24 h (**Figure 3A**), deucravacitinib inhibited IFNγ + IL-1β-induced apoptosis in a dose-dependent manner (60% and 92% protection at 10 and 1000 nmol/L, respectively) (**Figure 3L**). These results were confirmed by the caspase 3/7 activity (**Figure 3M**). Deucravacitinib did not affect IFNγ + IL-1β-induced STAT1 phosphorylation and protein expression (**Figure 3N**; **Supplementary Figures 3C, D**) or *HLA-ABC* mRNA expression (**Figure 3O**); in fact, 1000 nmol/L deucravacitinib increased P-STAT1 levels (**Supplementary Figure 3C**). Conversely, deucravacitinib diminished *MX1* and *CXCL10* mRNA expression, whereas *CHOP* was reduced only at 10 nmol/L deucravacitinib (**Figures 3P–R**).

### 3.5 The harmful effects of cytokines were partially inhibited by deucravacitinib

**Figure 4:**
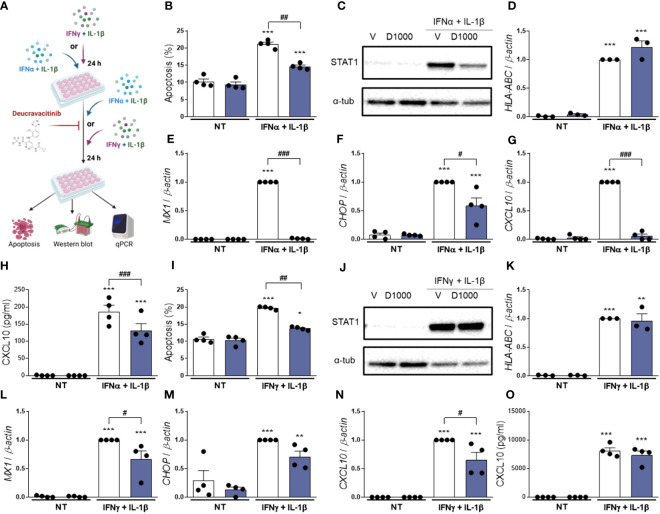
Treatment with deucravacitinib partially blocks IFNα + IL-1β- or IFNγ + IL-1β-induced changes. **(A)**: Experimental design of the pre-treatment with cytokines and subsequent exposure to IFNα + IL-1β or IFNγ + IL-1β in the presence of deucravacitinib for 24 h. EndoC-βH1 cells were left non-treated (NT) or pre-treated with IFNα + IL-1β (1000 U/mL + 50 U/mL, respectively) **(B–H)** or IFNγ + IL-1β (1000 U/mL + 50 U/mL, respectively) **(I–O)** for 24 h. Afterwards, cells were treated with vehicle (V, white bars) or 1000 nmol/L deucravacitinib (D1000, dark blue bars) in the absence (NT) or presence of IFNα + IL-1β or IFNγ + IL-1β for 24 h. **(B, I)**: Apoptosis was evaluated using Hoechst 33342/propidium iodide staining (*n* = 4 independent experiments). **(C, J)**: Representative immunoblots of STAT1 and α-tubulin (*n* = 4 independent experiments) **(D–G, K–N)**: mRNA expression of *HLA-ABC* **(D K)**, *MX1* **(E, L)**, *CHOP* **(F M)**, and *CXCL10* **(G, N)** was analyzed by real-time PCR, normalized to β-actin and then to the value of Vehicle treated with IFNα + IL-1β **(D–G)** or IFNγ + IL-1β **(K–N)** (considered as 1) (*n* = 3-4 independent experiments). **(H, O)**: CXCL10 secreted to the medium was determined by ELISA (*n* = 4 independent experiments). Data are mean ± SEM. *p ≤ 0.05, **p ≤ 0.01, ***p ≤ 0.001 vs. the respective non-treated (NT) (two-way ANOVA plus Sidak’s test). ^#^p ≤ 0.05, ^##^p ≤ 0.01, ^###^p ≤ 0.001, as indicated by bars (two-way ANOVA plus Dunnett’s test).

So far, we investigated whether pre-treatment with deucravacitinib prevents the effects of different cytokines in β-cells. Here, we assessed if deucravacitinib could abrogate these damaging effects. EndoC-βH1 cells were pre-treated with either IFNα + IL-1β or IFNγ + IL-1β for 24 h. Afterwards, 1000 nmol/L deucravacitinib was added for an additional 24 h still in the presence of cytokines (**Figure 4A**). Deucravacitinib partially decreased IFNα + IL-1β-induced apoptosis (60% decrease) (**Figure 4B**). IFNα + IL-1β-stimulated *HLA-ABC* mRNA expression remained unchanged in deucravacitinib-treated cells (**Figure 4D**), which agrees with previous data showing an IFNα-triggered long-lasting expression of *HLA-ABC* (13). STAT1 protein levels, CXCL10 secretion, and *CHOP* mRNA expression were reduced by 26-42% (**Figures 4C, F, H**; **Supplementary Figure 3E**), while the expression of *MX1* and *CXCL10* was completely inhibited by deucravacitinib (**Figures 4E, G**).

Similarly to IFNα + IL-1β, deucravacitinib diminished IFNγ + IL-1β-induced apoptosis (64% decrease) but did not modify *HLA-ABC* mRNA expression (**Figures 4I, K**). Protein levels of STAT1 and CXCL10, however, were not altered by TYK2 inhibition, whereas a slight, non-significant 30% reduction was seen in *CHOP* expression (**Figures 4J, M, O**; **Supplementary Figure 3F**). Expression of *MX1* and *CXCL10* was only partially affected by deucravacitinib under IFNγ + IL-1β conditions (**Figures 4L, N**).

## 4 Discussion

Targeting the JAK-STAT pathway has emerged as a promising therapeutic approach for type 1 diabetes prevention/early treatment (3, 15). Although this strategy has been approved for treatment of some autoimmune diseases, including rheumatoid arthritis and psoriatic arthritis (37), there are no JAK inhibitors approved for type 1 diabetes. Nonetheless, recent preclinical data suggest that these inhibitors could be repurposed for this disease (13, 14, 16, 17, 22, 23, 38) and a clinical trial investigating whether baricitinib prevents the progressive, immune-mediated destruction of β-cells in type 1 diabetes patients is ongoing (39).

In the current study, we tested whether the TYK2 inhibitor deucravacitinib could protect human β-cells against the deleterious effects of IFNα and other cytokines. We focused on this TYK2 inhibitor for two reasons: first, due to TYK2 importance for type 1 diabetes pathogenesis. For instance, TYK2 regulates IFNα-mediated pro-apoptotic and proinflammatory pathways in β-cells (21, 22). Second, exploring a drug recently approved by the U.S. Food and Drug Administration to treat another autoimmune disease, namely plaque psoriasis (26), increases its repositioning potential for type 1 diabetes and facilitates the bench-to-bedside transition.

Deucravacitinib is a small-molecule ligand that binds to and stabilizes the TYK2 pseudokinase domain, leading to highly potent and selective allosteric TYK2 inhibition (24, 40). Inhibition of IFNα-induced STAT phosphorylation by deucravacitinib has been shown in several cell types, such as CD3^+^ T cells, CD19^+^ B cells, and CD14^+^ monocytes (24). Here we showed that deucravacitinib also prevents IFNα-stimulated STAT1 and STAT2 phosphorylation in human EndoC-βH1 cell line. Furthermore, in agreement with previous findings (24), deucravacitinib also showed higher potency against TYK2-mediated phosphorylation of STAT1 compared with STAT2 phosphorylation in our experimental model. Notably, at the concentrations used in our study, deucravacitinib did not affect β-cell function and viability, which is a desired feature for a drug with therapeutic potential.

Compared with ruxolitinib and baricitinib, two clinically available JAK1/JAK2 inhibitors, deucravacitinib was more potent against IFNα-stimulated STAT phosphorylation, ISRE activity, and mRNA expression of *HLA-ABC*, *MX1*, and *CXCL10*. However, unlike ruxolitinib and baricitinib, deucravacitinib did not affect the IFNα-mediated upregulation of the ER stress markers *CHOP* and *XBP1s*. Our results partially agree with a previous publication reporting that two TYK2 inhibitors failed to prevent IFNα-induced *CHOP* expression in EndoC-βH1 cells (23). Prior studies have shown that other JAK/TYK2 inhibitors could prevent the detrimental effects of IFNα + IL-1β, such as apoptosis and inflammation (14, 23). Therefore, we investigated whether deucravacitinib could protect β-cells against the harmful effects of two different combinations of cytokines: IFNα + IL-1β (early insulitis) and IFNγ + IL-1β (late insulitis). In both scenarios, pre-treatment with deucravacitinib protected against cytokine-induced apoptosis and *CXCL10* mRNA expression. Additionally, in cells treated with IFNα + IL-1β, pre-treatment with deucravacitinib blocked the overexpression of MHC class I at the cell surface and CXCL10 secretion to the medium. Interestingly, while the IFNα + IL-1β-induced upregulation of *HLA-ABC*, *MX1*, and *CHOP* was inhibited by the pre-treatment with deucravacitinib, this inhibitor did not change the expression of *HLA-ABC* stimulated by IFNγ + IL-1β. Moreover, *MX1* and *CHOP* mRNA expression was only partially reduced by the pre-treatment with deucravacitinib in IFNγ + IL-1β-treated cells. Importantly, the addition of deucravacitinib when cytokine exposure was already ongoing could reduce the deleterious effects of these cytokines. Although it seems clear that deucravacitinib confers protection against IFNα + IL-1β by directly inhibiting the TYK2-mediated pathway, it remains to be answered how deucravacitinib protects against IFNγ + IL-1β-induced effects. Indeed, our present data suggest that deucravacitinib does not interfere with the IFNγ-mediated signaling pathway. One possibility might be the following: in β-cells, either IFNγ alone or in combination with IL-1β induce the expression of members of the interferon regulatory factor (IRF) family, such as IRF3 and IRF7 (41, 42). As IRF3 and IRF7 are potent activators of IFNα and IFNβ gene expression (43, 44), it is conceivable that IFNγ + IL-1β-induced IRF3 and IRF7 could lead to type I IFN expression and secretion. Then, secreted IFNα and/or IFNβ could stimulate the type I IFN receptor-TYK2 pathway in an autocrine fashion. In this context, deucravacitinib could inhibit this positive-feedback loop stimulated by IFNγ + IL-1β-induced IRF3 and IRF7 expression.

Based on our findings, it will be interesting to test whether novel small molecule TYK2 pseudokinase ligands (45) could also protect β-cells from IFNα deleterious effects. Nevertheless, we must bear in mind that completely inhibiting TYK2 may be counterproductive, as it might lead to susceptibility to microorganisms (e.g., mycobacteria and virus) and immunodeficiency (46). Thus, regardless of the TYK2 inhibitor chosen, we should focus on doses that induce a partial inhibition, as seen in individuals with a protective single nucleotide polymorphism in the *TYK2* gene (18), as it could offer maximal efficacy with reduced risk of developing secondary infections. Moreover, our data suggest that partial TYK2 inhibition obtained with low doses of deucravacitinib was enough to prevent most IFNα-induced harmful effects in β-cells, such as upregulation of the pro-apoptotic *CHOP*, MHC class I overexpression, and apoptosis (in the presence of IL-1β). One potential limitation of our study is its purely *in vitro* nature, which may limit our conclusions regarding the use of deucravacitinib to treat a disease as complex as type 1 diabetes. Conversely, our findings, along with others (22, 23), provide further preclinical evidence that TYK2 inhibitors could be considered a strategy for an early therapy for type 1 diabetes. The next logical step would be to investigate whether our *in vitro* findings could be translated to animal models of type 1 diabetes (e.g., NOD and RIP-B7.1 mice).

In conclusion, we provided evidence that deucravacitinib protects β-cells against the deleterious effects of proinflammatory cytokines, such as IFNα, IFNγ and IL-1β, without affecting β-cell function and survival. Our present findings add to the existing evidence that TYK2 inhibition may be an efficient treatment strategy for type 1 diabetes. Moreover, these preclinical findings suggest that deucravacitinib could be repurposed to treat pre-symptomatic type 1 diabetes subjects (i.e., positive for 2–3 autoantibodies but still normoglycemic) or be introduced in the early stages of type 1 diabetes onset.

## Data availability statement

The raw data supporting the conclusions of this article will be made available by the authors, without undue reservation.

## Ethics statement

Ethical approval was not required for the studies on humans in accordance with the local legislation and institutional requirements because only commercially available established cell lines were used.

## Author contributions

RS: Conceptualization, Data curation, Formal Analysis, Investigation, Methodology, Supervision, Visualization, Writing – original draft, Writing – review & editing. DG-L: Formal Analysis, Investigation, Writing – review & editing. AP-S: Formal Analysis, Investigation, Writing – review & editing. AN: Resources, Writing – review & editing. LM: Conceptualization, Data curation, Formal Analysis, Funding acquisition, Investigation, Methodology, Project administration, Resources, Supervision, Visualization, Writing – original draft, Writing – review & editing.
