# Supplementary material for: Deucravacitinib, a tyrosine kinase 2 pseudokinase inhibitor, protects human EndoC-βH1 β-cells against proinflammatory insults
Source: Front Immunol. 2023 Oct 3;14:1263926. doi: 10.3389/fimmu.2023.1263926 (PMC10579912; doi:10.3389/fimmu.2023.1263926)

Supplementary Material for

Deucravacitinib, a tyrosine kinase 2 pseudokinase inhibitor, protects human EndoC-βH1 β-cells against proinflammatory insults

Supplementary Methods

Glucose-stimulated insulin secretion

Insulin secretion: EndoC-βH1 cells (70,000 cells/well) were incubated in modified Krebs-Ringer buffer (115 mmol/L NaCl, 24 mmol/L $NaHCO_3$, 5 mmol/L KCl, 1 mmol/L $CaCl_2$, 1 mmol/L $MgCl_2$, 10 mmol/L HEPES pH 7.4, and 0.1% BSA) for 1 h before glucose stimulation. Upon starvation, cells were sequentially incubated with low (0 mmol/L) and high glucose (20 mmol/L) for 1 h (each incubation). After each stimulatory period, the incubation medium was collected, placed onto ice, and centrifuged for 5 min at 700 *g* (4°C). The supernatant was transferred into a new, fresh tube and stored at -20°C until insulin measurements. The amount of secreted insulin was calculated as *% of total insulin* and data were normalized to insulin secretion at 20 mmol/L glucose in vehicle-treated cells without IFNα (considered as 100%).

Insulin content: upon incubation with low and high glucose, cells were lysed in a cell lysis solution containing 137 mmol/L NaCl, 0.1% Triton X100, 1% glycerol, 2 mmol/L EGTA, 20 mmol/L Tris pH 8.0, and protease inhibitor cocktail. Cell lysates were centrifuged for 5 min at 700 *g* (4°C); the supernatant was transferred into a new, fresh tube and stored at -20°C until insulin measurements. Insulin content (ng insulin/10^6 cells) was normalized to the condition Vehicle non-treated (NT) (considered as 100%).

Insulin secreted and insulin content from lysed cells were measured using a human insulin ELISA kit (Mercodia, Uppsala, Sweden).

	**Forward**	**Reverse**
	**Sequence (5’-3’)**	**Sequence (5’-3’)**
β-actin	CTGTACGCCAACACAGTGCT	GCTCAGGAGGAGCAATGATC
HLA-ABC	GAGAACGGGAAGGAGACGC	CATCTCAGGGTGAGGGGCT
CXCL10	GTGGCATTCAAGGAGTACCTC	GCCTTCGATTCTGGATTCAG
MX1	AGACAGGACCATCGGAATCT	GTAACCCTTCTTCAGGTGGAAC
ATF3	GCTGTCACCACGTGCAGTAT	TTTGTGTTAACGCTGGGAGA
CHOP	AACGGAAACAGAGTGGTCATT	GCTTGAGCCGTTCATTCTCT
XBP1s	CCGCAGCAGGTGCAGG	GAGTCAATACCGCCAGAATCCA

Supplementary Table 1. Primers used in the present study.

Antibody	Manufacturer	Catalogue number	Species raised in	Dilution	RRID
Phospho-STAT1	Cell Signaling Technology	9167	Rabbit, monoclonal	1:1000	AB_561284
Phospho-STAT2	Cell Signaling Technology	88410	Rabbit, monoclonal	1:1000	AB_2800123
STAT1	Cell Signaling Technology	9172	Rabbit, polyclonal	1:1000	AB_2198300
STAT2	Santa Cruz Biotechnology	sc-514193	Mouse, monoclonal	1:500	AB_2810271
MHC Class I (W6/32)	Enzo Life Sciences	ALX-805-711-C100	Mouse, monoclonal	1:1000	AB_11179235
α-Tubulin	Sigma	T9026	Mouse, monoclonal	1:5000	AB_477593
Goat anti-mouse IgG	Bio-rad	170-6516	Goat, polyclonal	1:5000	AB_11125547
Goat anti-rabbit IgG	Bio-rad	170-6515	Goat, polyclonal	1:5000	AB_11125142
Alexa Fluor 568 goat anti-mouse IgG	Invitrogen	A-11031	Goat, polyclonal	1:500	AB_144696

Supplementary Table 2. Antibodies used in the present study.

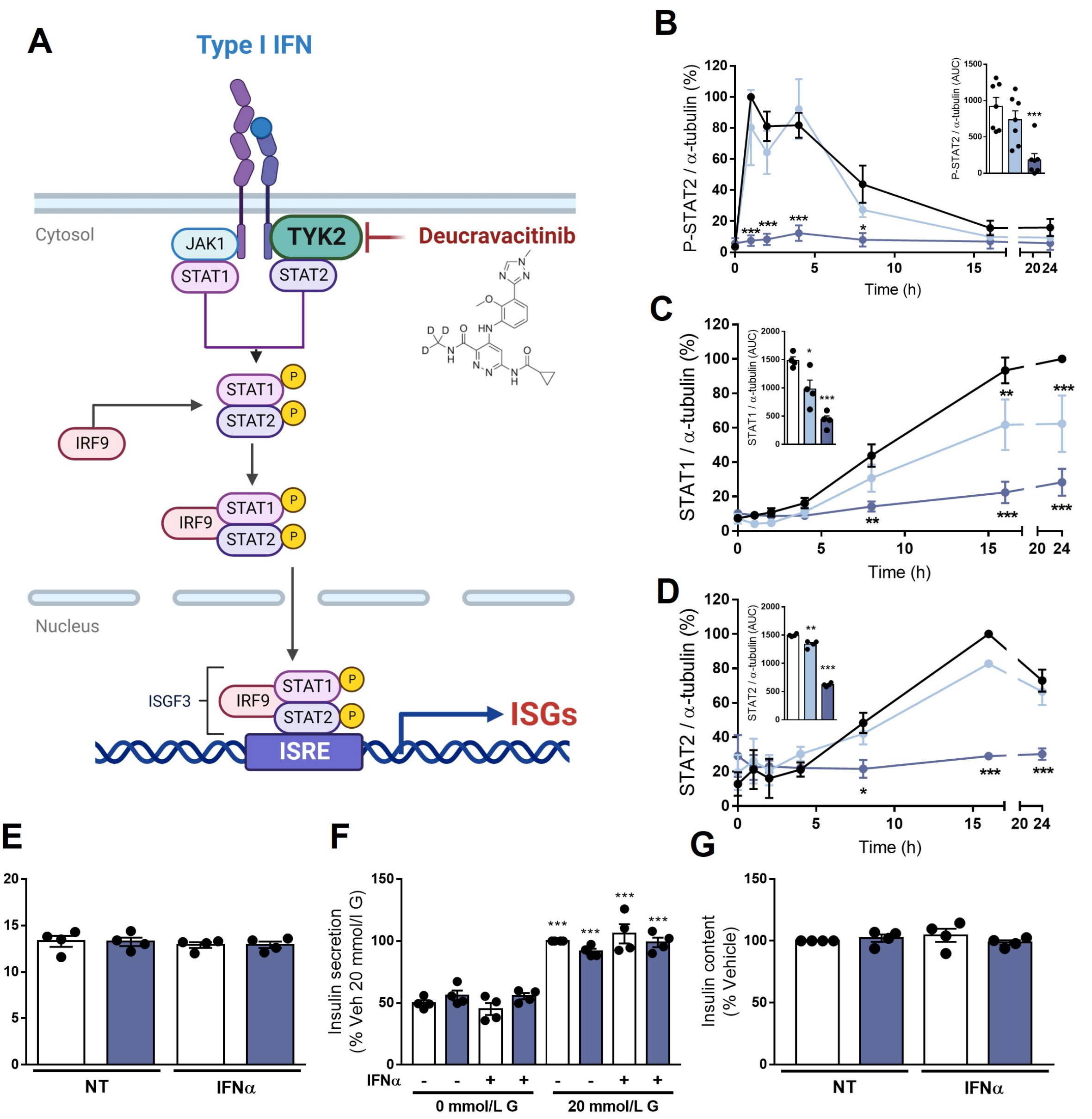

Supplementary Figure 1. Deucravacitinib prevents IFNα-induced effects without affecting β-cell survival and function. *A*: Schematic representation of the type I IFN signaling pathway. IFNα binding to its receptor, namely IFNα receptor, is followed by the activation of JAK1 and TYK2. In turn, JAK1 and TYK2 promote the phosphorylation and activation of STAT1 and STAT2. Once activated, P-STAT1 and P-STAT2 dimerize and associate with the Interferon Regulatory Factor 9 (IRF9) to form a transcriptional complex known as IFN-stimulated gene factor 3 (ISGF3). ISGF3 is imported to the nucleus, where it binds to specific elements named IFN-stimulated regulatory element (ISRE); ISGF3 binding to ISRE initiates the transcription of IFN-stimulated genes (ISGs). Deucravacitinib is a small-molecule ligand that binds to and stabilizes the TYK2 pseudokinase domain, leading to highly potent and selective allosteric TYK2 inhibition. *B-G*: EndoC-βH1 cells were treated with vehicle (V or Veh, black circles or white bars) or pre-treated with deucravacitinib (10 [D10, soft blue circles] and 1000 nmol/L [D1000, dark blue circles or bars]) for 1 h. Afterwards, cells were left non-treated or treated with IFNα (1000 U/mL) in the absence or presence of deucravacitinib for 1–24 h (*B-D*) or 24 h (*E-G*). *B-D*: Quantification of P-STAT2 (*B*), STAT1 (*C*), and STAT2 (*D*) from immunoblots shown in Fig. 1C. The insets in *B*, *C*, and *D* are the area under curve (AUC)

of P-STAT2, STAT1, and STAT2, respectively. Values were normalized to α-tubulin, and then to the highest value of each experiment (considered as 100%) (*n* = 3-7 independent experiments). *E*: Apoptosis was evaluated using Hoechst 33342/propidium iodide staining (*n* = 4 independent experiments). *F* and *G*: Glucose-stimulated insulin secretion (*F*) and insulin content (*G*) were measured by ELISA. Data are mean ± SEM. *B*: ***$p \leq 0.001$ vs. Vehicle + IFNα (two-way ANOVA plus Dunnett's test). Insets in *B*-*D*: **$p \leq 0.01$, ***$p \leq 0.001$ vs. Vehicle + IFNα (one-way ANOVA plus Dunnett's test). *F*: ***$p \leq 0.001$ vs. the respective treatment at 0 mmol/L glucose (G) (two-way ANOVA plus Sidak's test).

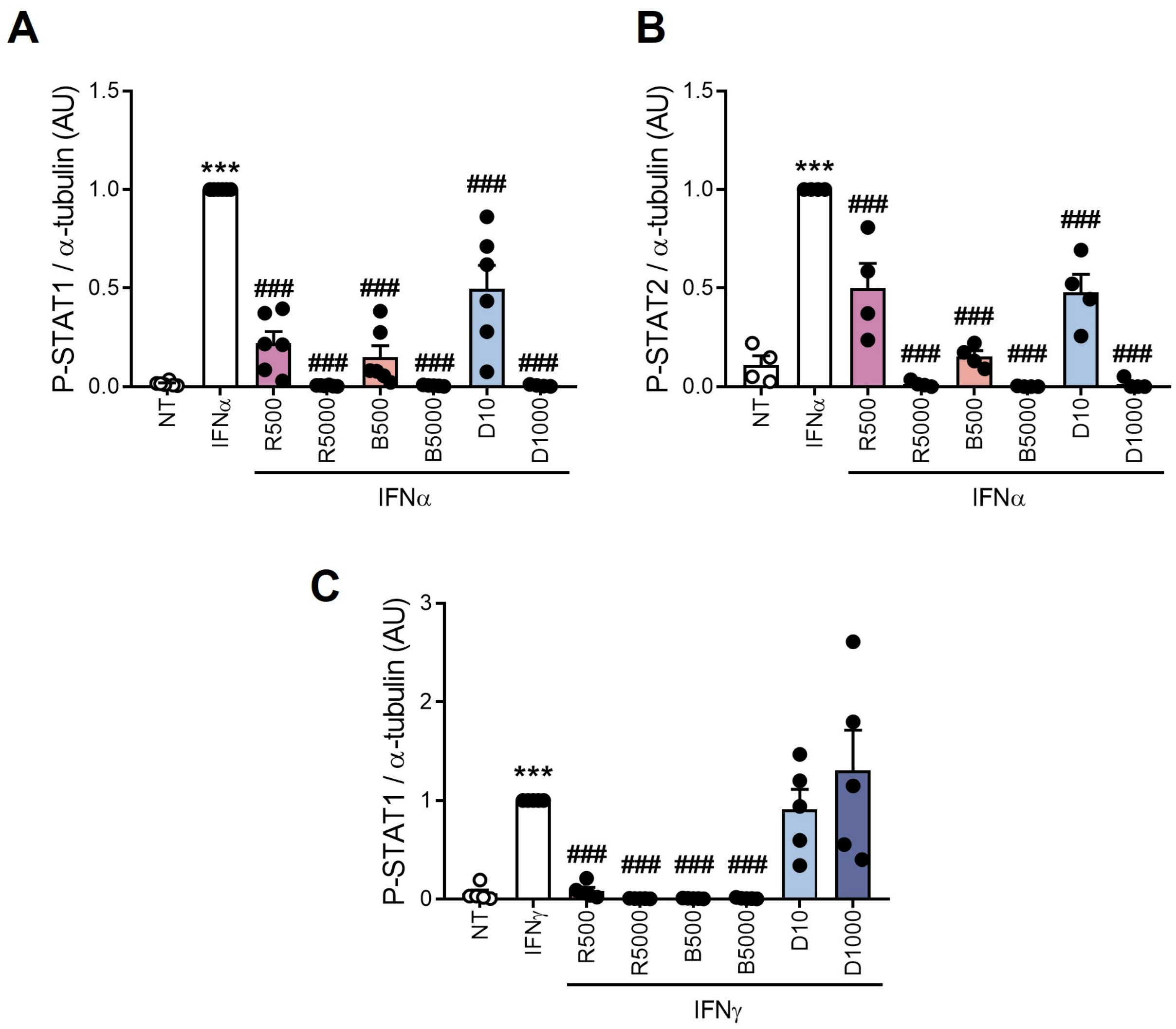

Supplementary Figure 2. JAK/TYK2 inhibitors prevent STAT1 and STAT2 phosphorylation. *A-C*: EndoC-βH1 cells were treated with vehicle (V, white bars) or pre-treated with ruxolitinib (500 and 5000 nmol/L; R500 and R5000), baricitinib (500 and 5000 nmol/L; B500 and B5000), or deucravacitinib (10 and 1000 nmol/L; D10 and D1000) for 1 h. After the pre-treatment, cells were left non-treated (NT, white circles) or treated with either IFNα (1000 U/mL) (*A* and *B*) or IFNγ (1000 U/mL) (*C*) in the absence or presence of each inhibitor for 1 h. Quantification of P-STAT1 (*A* and *C*) and P-STAT2 (*B*) from immunoblots shown in Fig. 2B and C, respectively. Values were normalized to α-tubulin, and then to the highest value of each experiment (considered as 1) (*n* = 4-6 independent experiments). Data are mean ± SEM. ***p≤0.001 vs. the respective non-treated (NT) (one-way ANOVA plus Dunnett's test). ###p≤0.001 vs. IFNα (*A* and *B*) or IFNγ (*C*) (one-way ANOVA plus Dunnett's test).

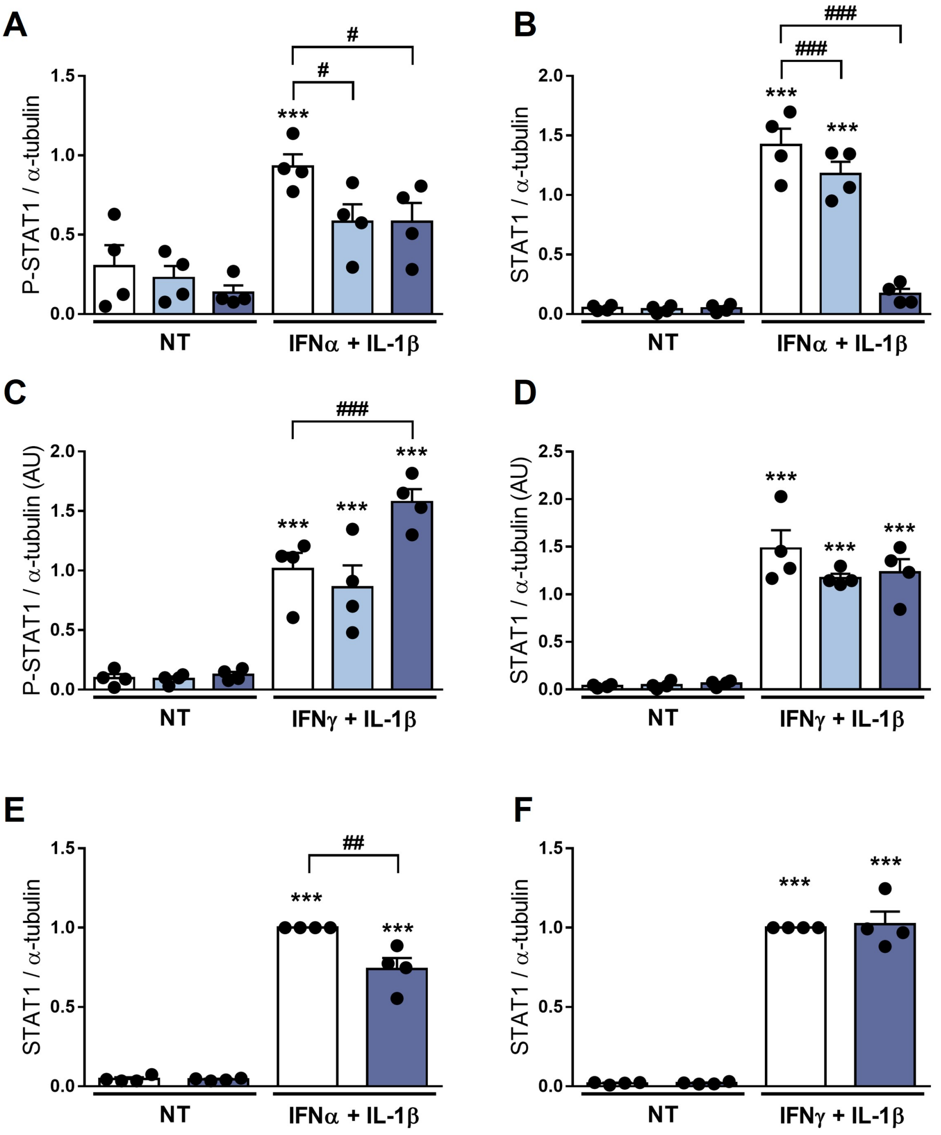

Supplementary Figure 3. Deucravacitinib inhibits IFNα + IL-1β- but not IFNγ + IL-1β-induced STAT1 phosphorylation and expression. *A-D*: EndoC-βH1 cells were treated with vehicle (V, white bars) or pre-treated with deucravacitinib (10 [D10, soft blue bars] and 1000 nmol/L [D1000, dark blue bars]) for 1 h. Afterwards, cells were left non-treated (NT) or treated with IFNα + IL-1β (1000 U/mL + 50 U/mL, respectively) (*A* and *B*) or IFNγ + IL-1β (1000 U/mL + 50 U/mL, respectively) (*C* and *D*) in the absence or presence of deucravacitinib for 24 h. Quantification of P-STAT1 (*A* and *C*) and STAT1 (*B* and *D*) from immunoblots shown in Fig. 3D and N, respectively. Values were normalized to α-tubulin, and then to the highest value of each experiment (considered as 1) (n = 4 independent experiments). *E* and *D*: EndoC-βH1 cells were left non-treated (NT) or pre-treated with IFNα + IL-1β (1000 U/mL + 50 U/mL, respectively) (*E*) or IFNγ + IL-1β (1000 U/mL + 50 U/mL, respectively) (*F*) for 24 h. Afterwards, cells were treated with vehicle (V, white bars) or 1000 nmol/L deucravacitinib (D1000, dark blue bars) in the absence (NT) or presence of IFNα + IL-1β or IFNγ + IL-1β for 24 h. Quantification of STAT1 from immunoblots shown in Fig. 3D and N, respectively. Values were normalized to α-tubulin, and then to the highest value of each experiment (considered as 1) (n = 4 independent experiments). Data are mean ± SEM. ***$p \leq 0.001$ vs. the respective non-treated (NT) (two-way ANOVA plus Sidak's test). $^{\#}p \leq 0.05$, $^{\#\#}p \leq 0.01$, $^{\#\#\#}p \leq 0.001$, as indicated by bars (two-way ANOVA plus Dunnett's test).

Original, uncropped images representing immunoblots and microscopic photos

Figure 1A

Full unedited blot for **Figure 1A**

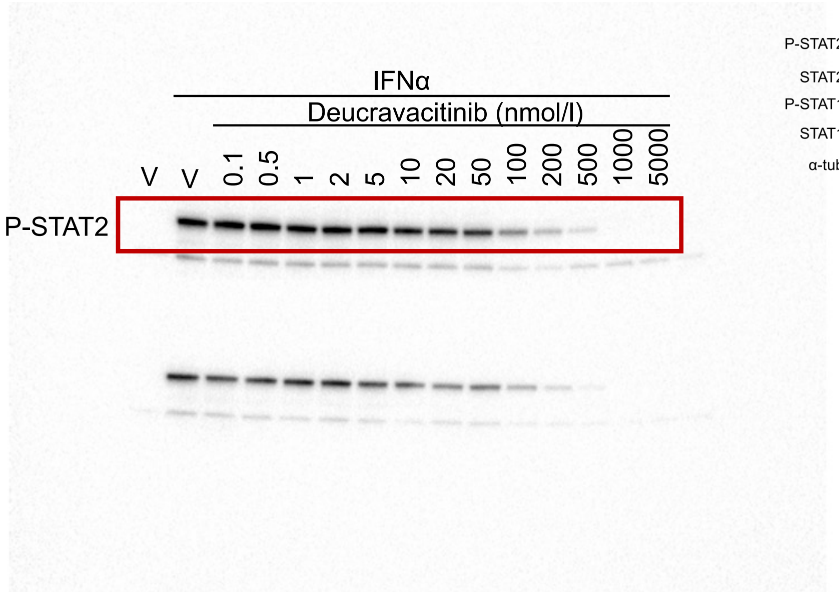

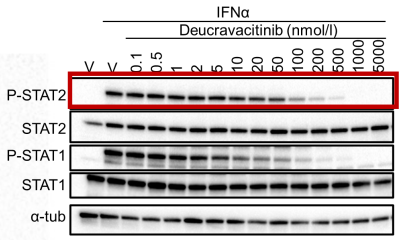

Full unedited blot for **Figure 1A**

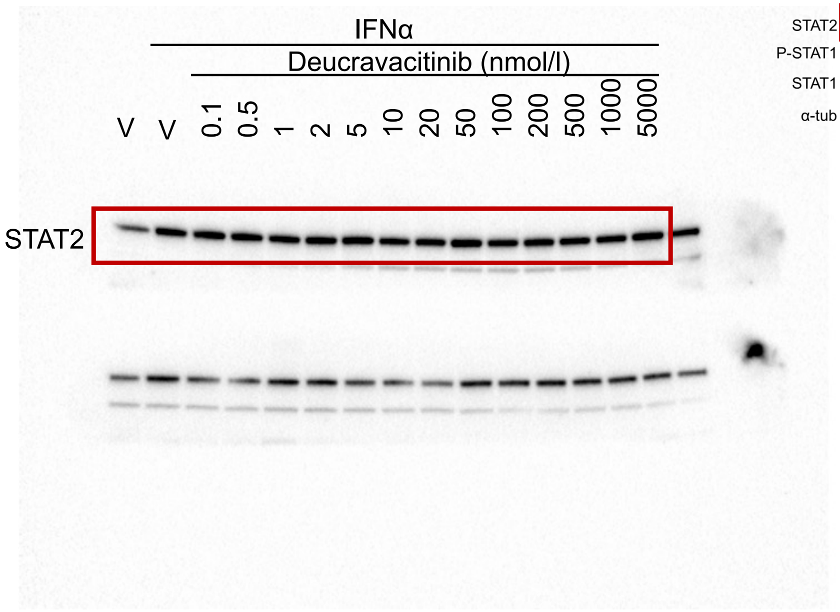

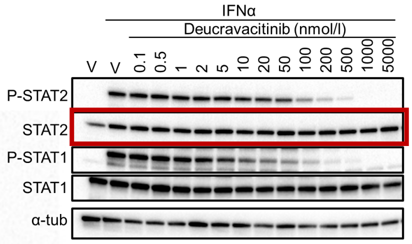

Full unedited blot for **Figure 1A**

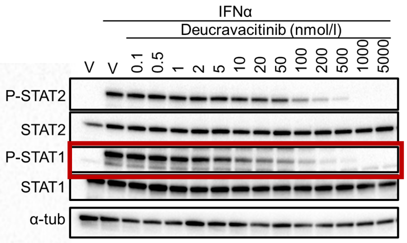

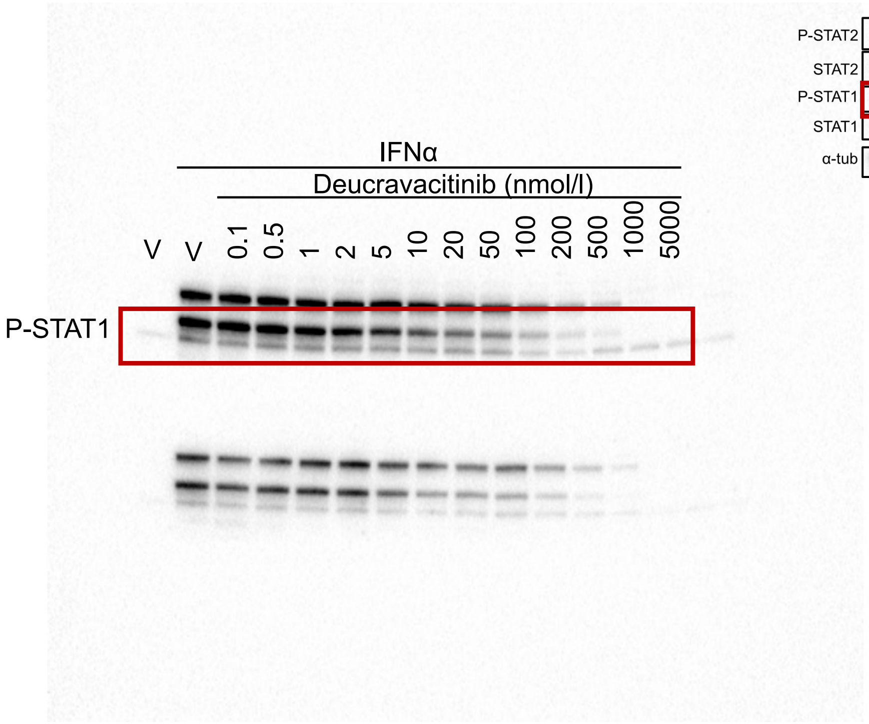

Full unedited blot for **Figure 1A**

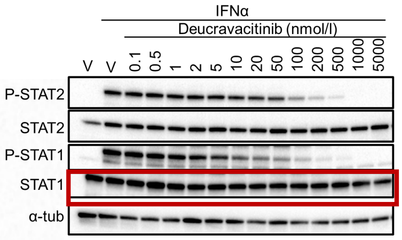

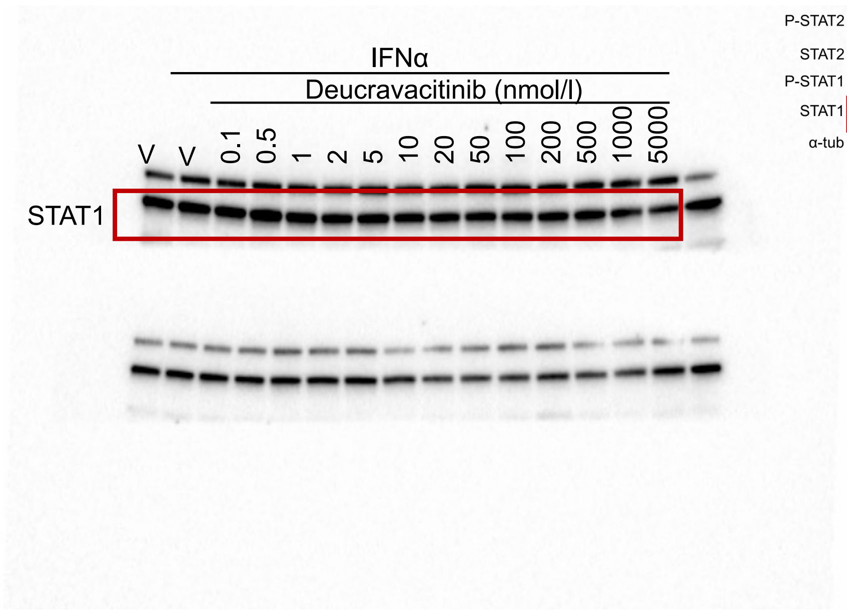

Full unedited blot for **Figure 1A**

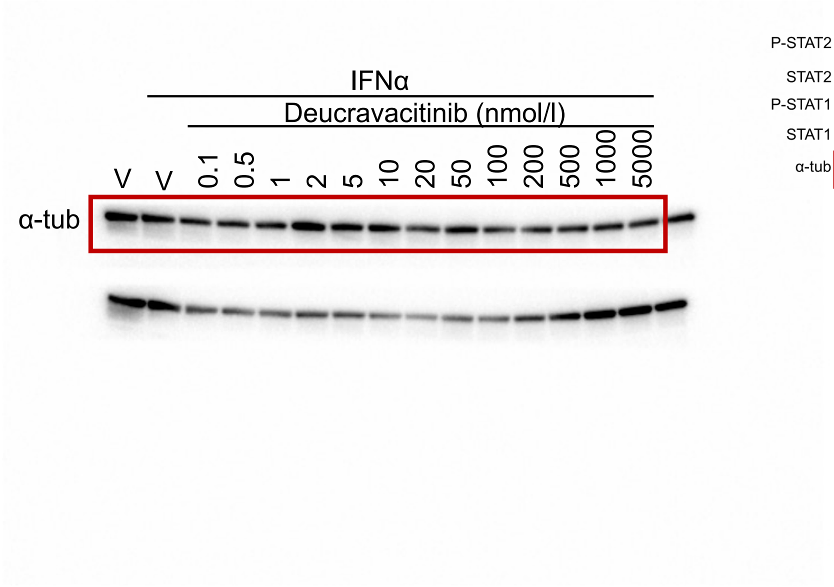

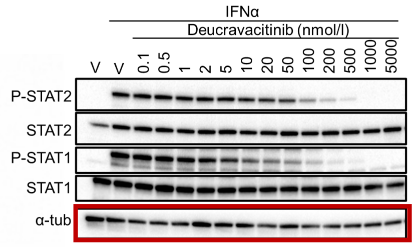

Figure 1C

Full unedited blot for **Figure 1C**

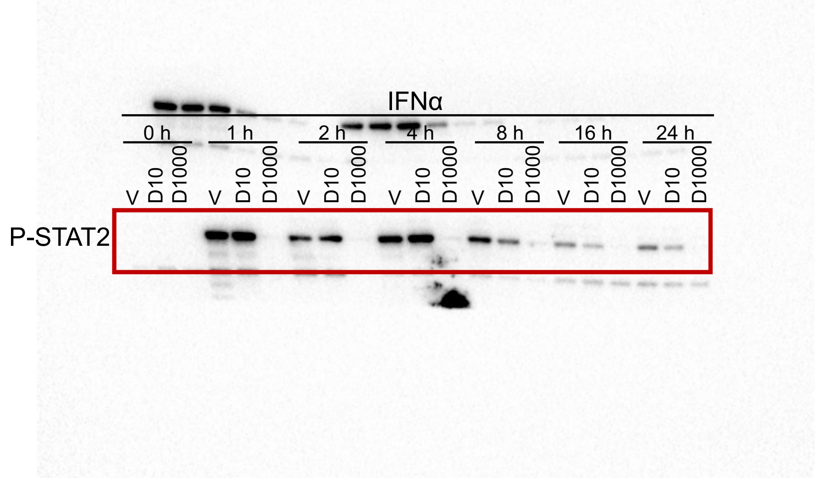

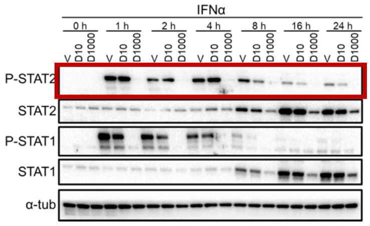

Full unedited blot for **Figure 1C**

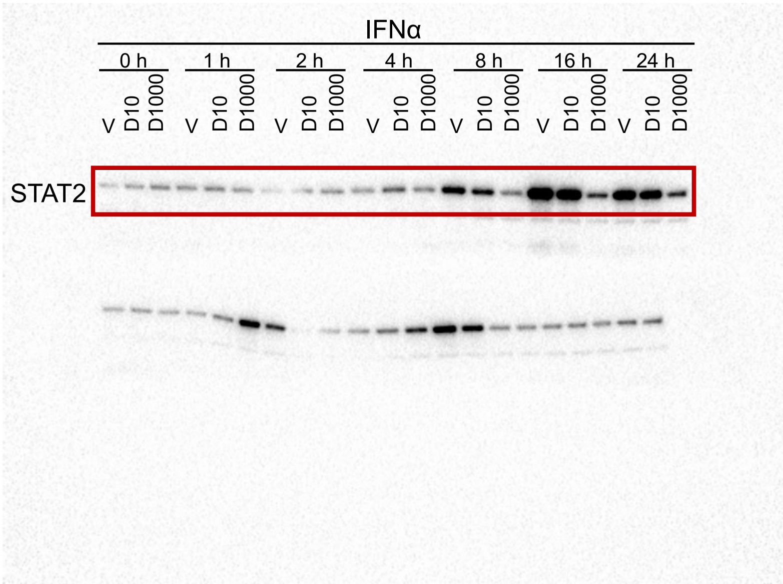

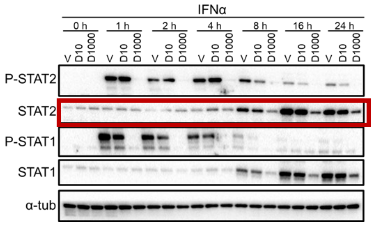

Full unedited blot for **Figure 1C**

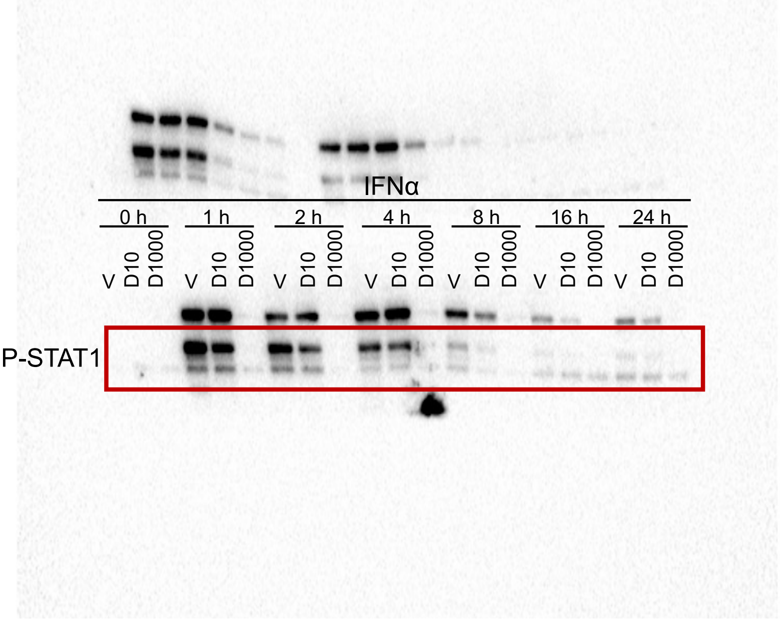

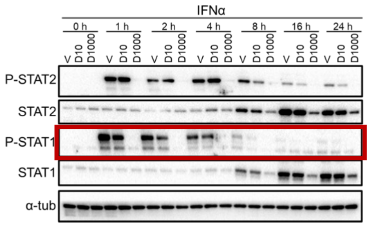

Full unedited blot for **Figure 1C**

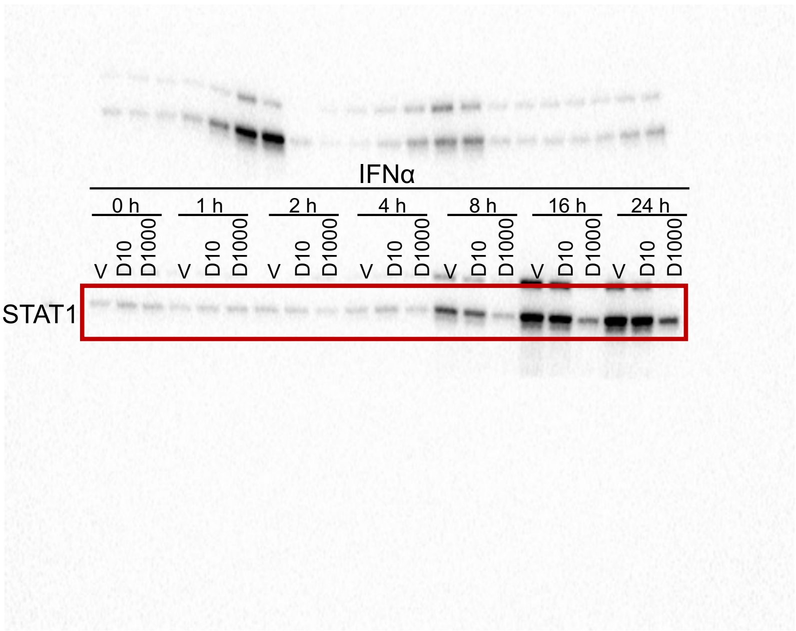

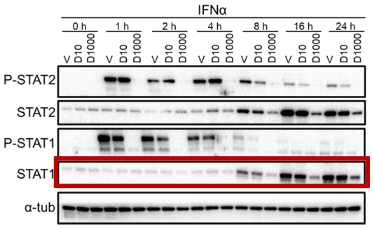

Full unedited blot for **Figure 1C**

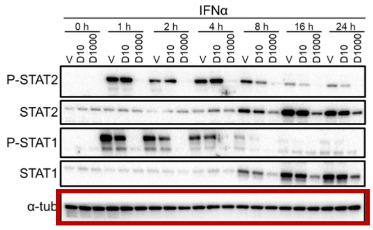

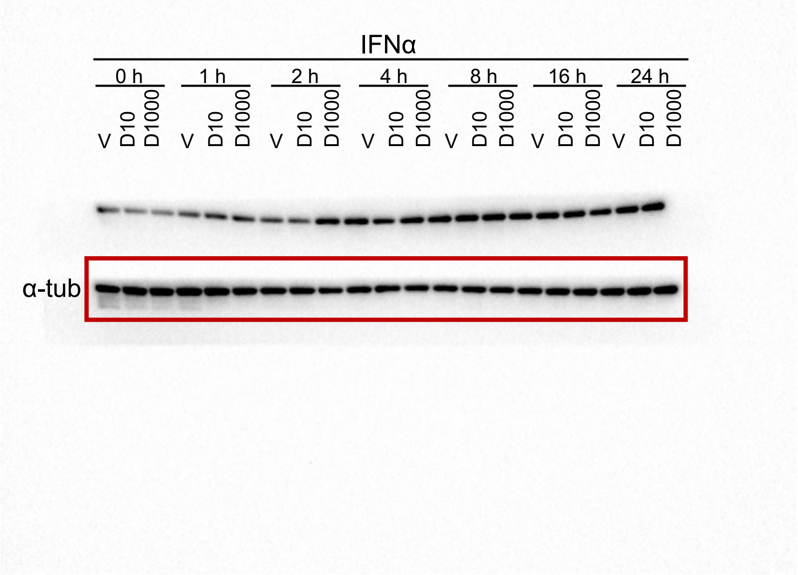

Figure 1E

Full unedited photo for **Figure 1E**

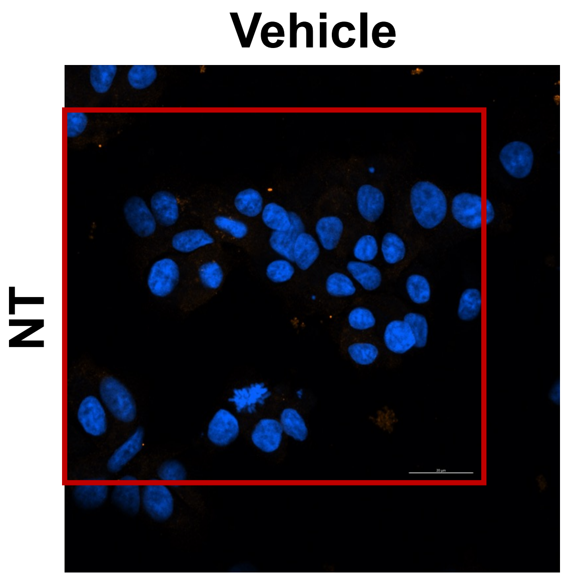

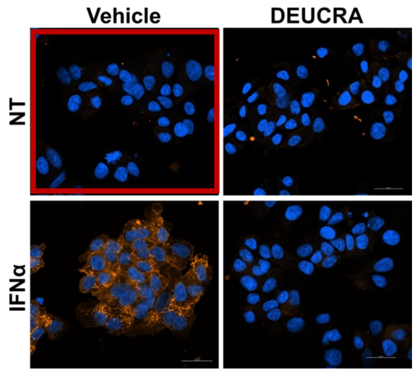

Full unedited photo for **Figure 1E**

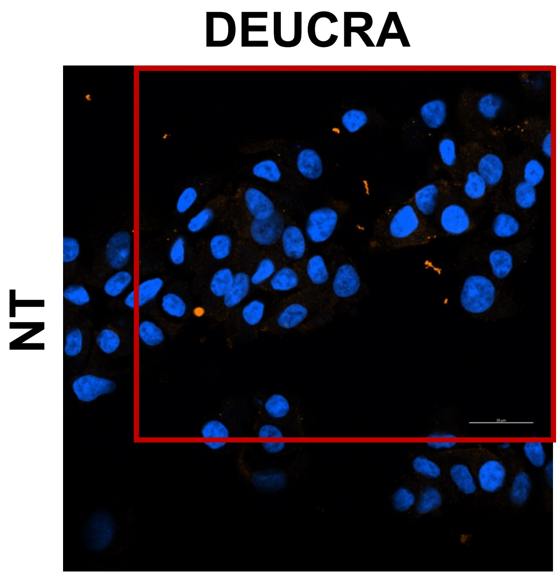

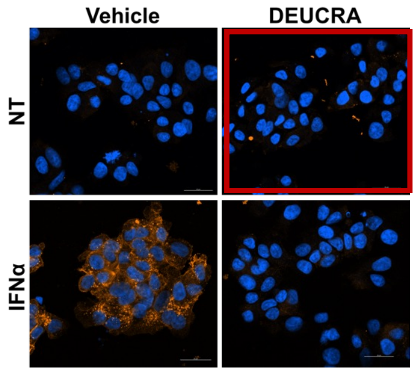

Full unedited photo for **Figure 1E**

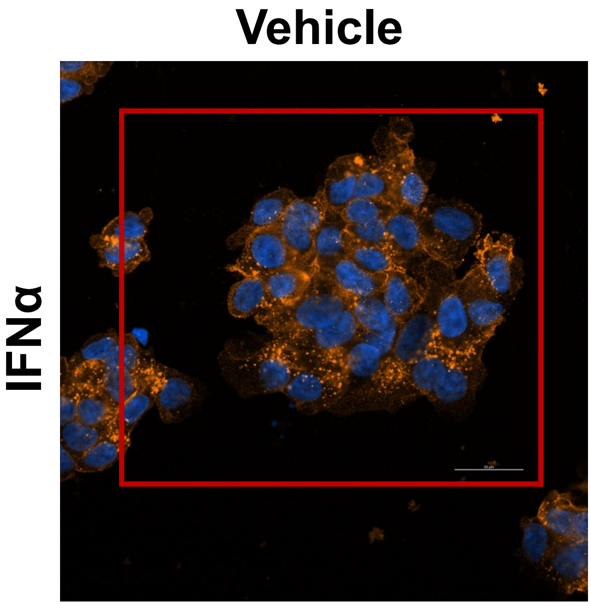

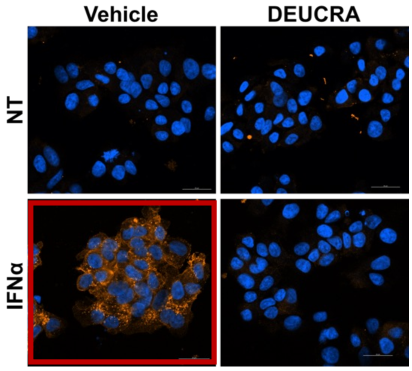

Full unedited photo for **Figure 1E**

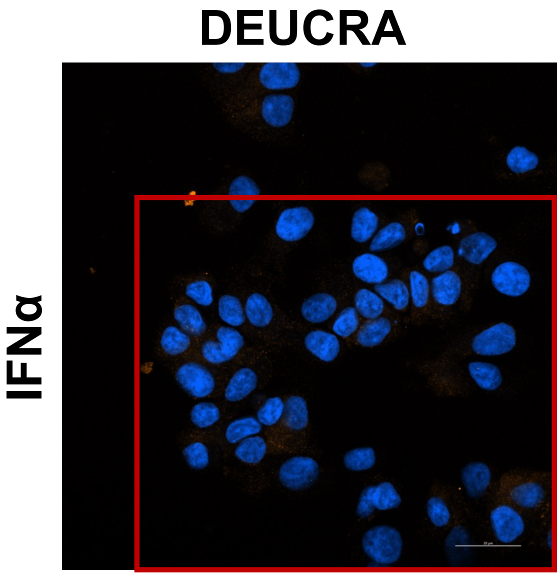

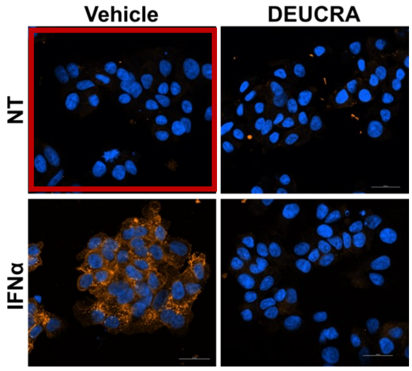

Figure 2B

Full unedited blot for **Figure 2B**

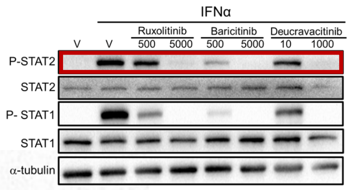

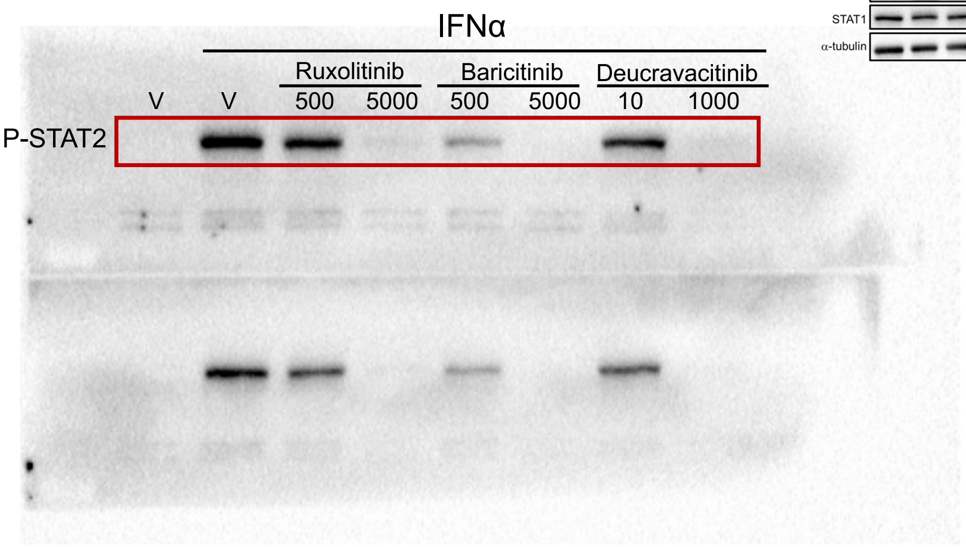

Full unedited blot for **Figure 2B**

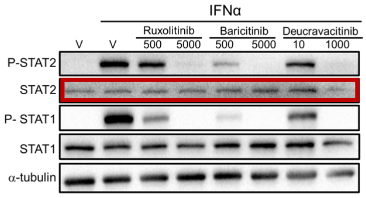

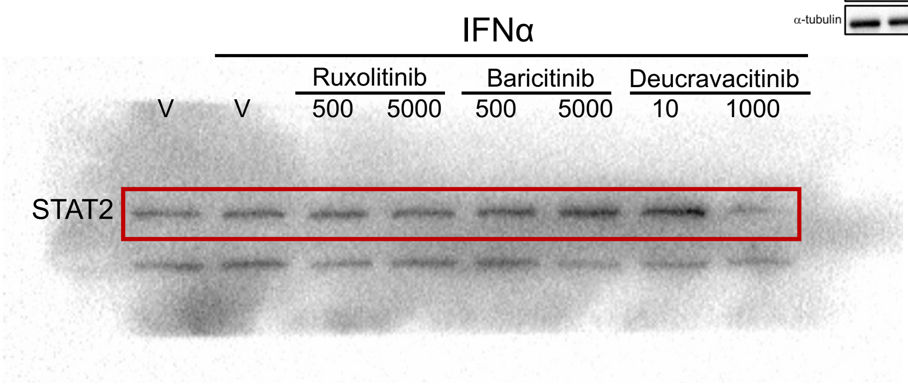

Full unedited blot for **Figure 2B**

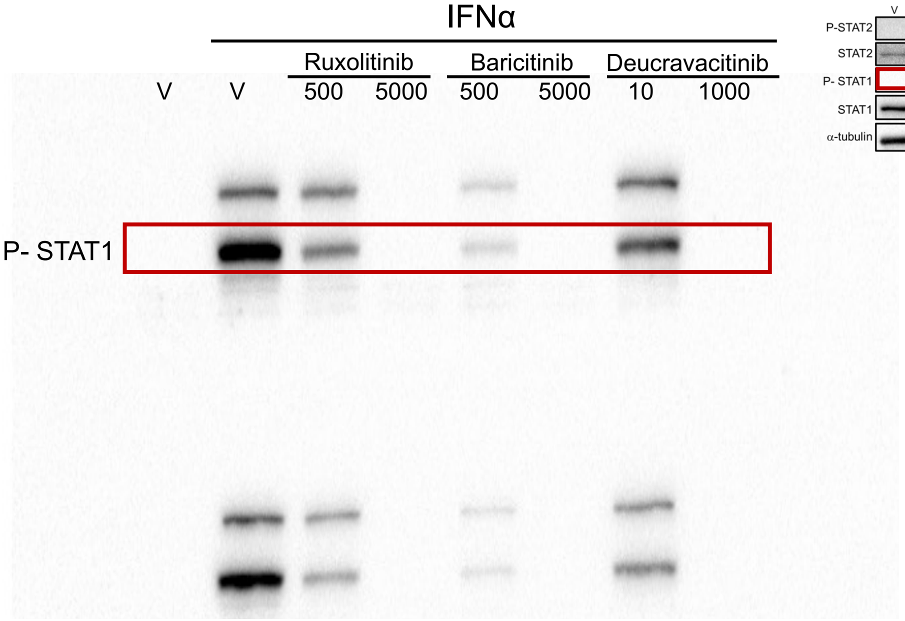

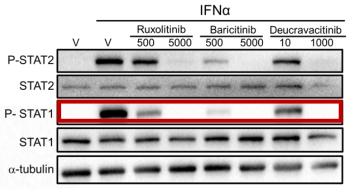

Full unedited blot for **Figure 2B**

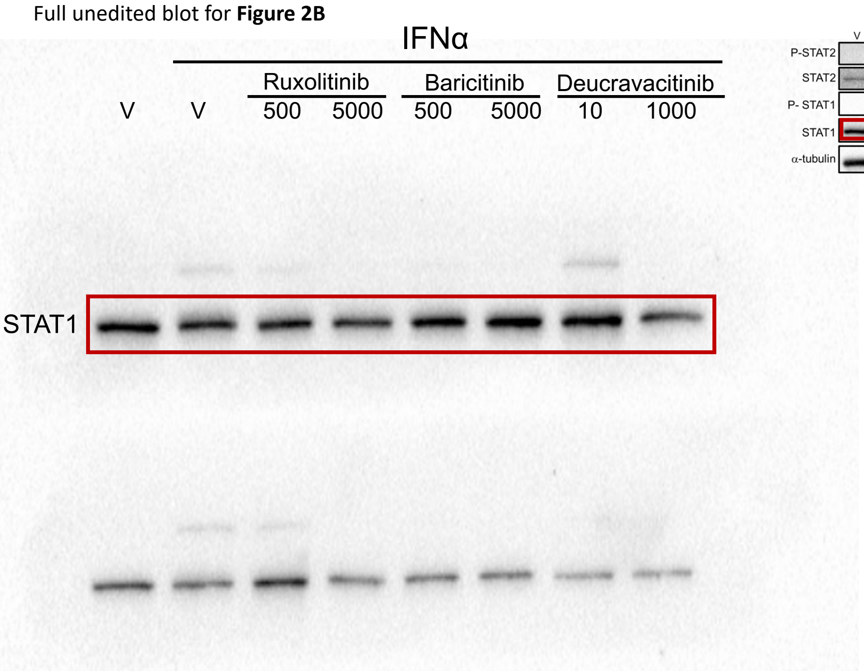

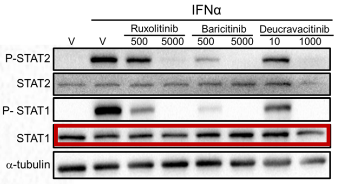

Full unedited blot for **Figure 2B**

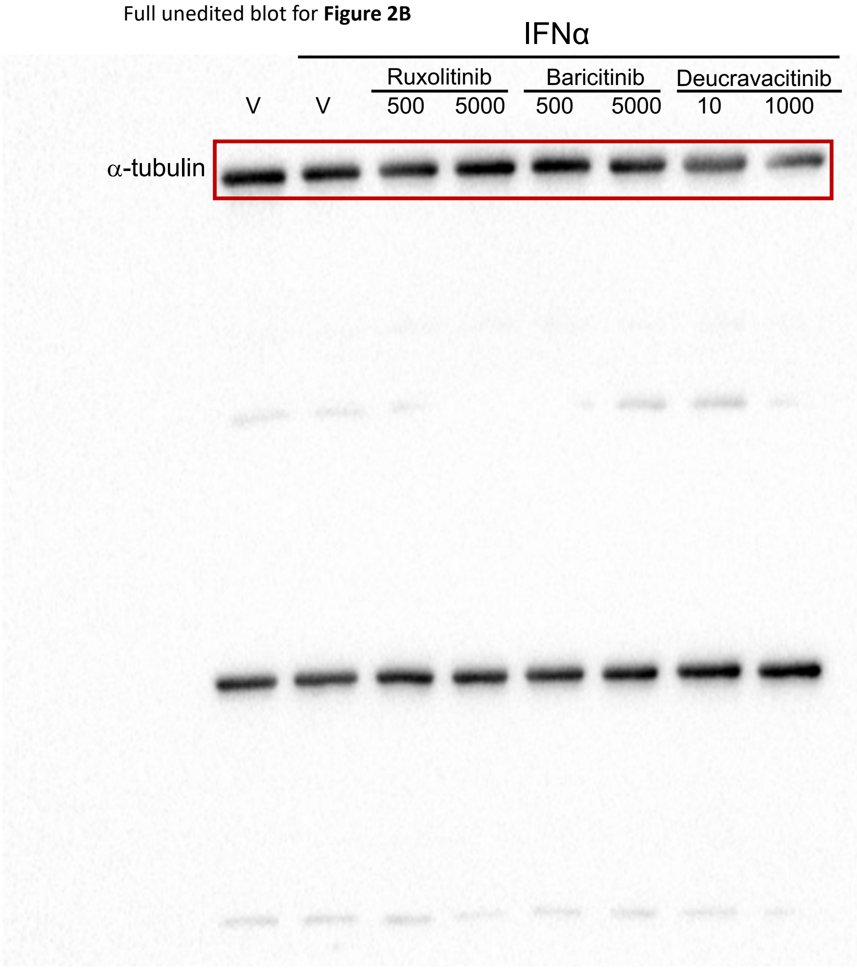

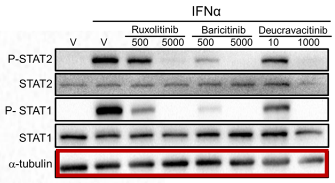

Figure 2C

Full unedited blot for **Figure 2C**

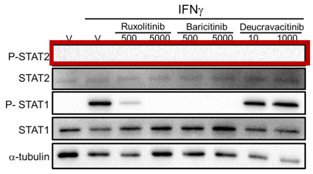

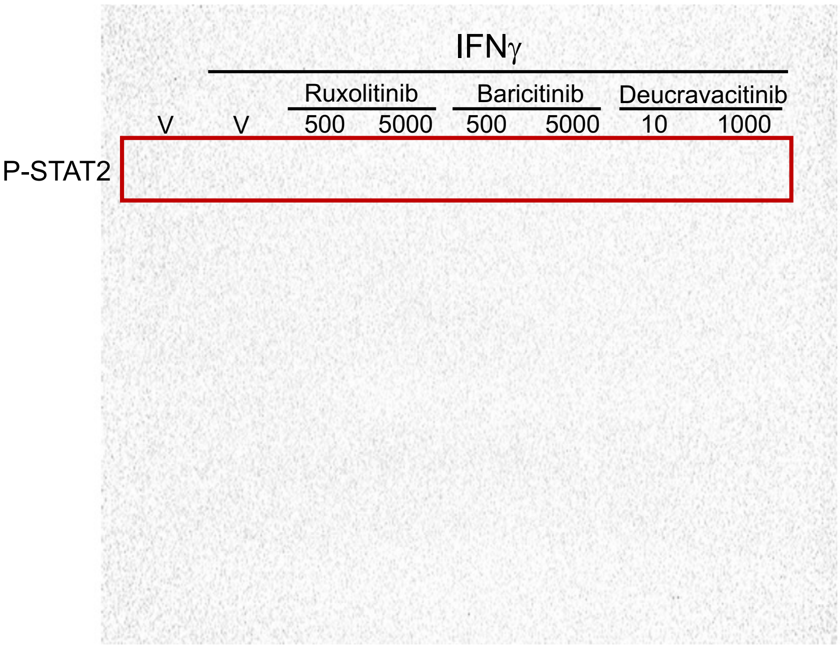

Full unedited blot for **Figure 2C**

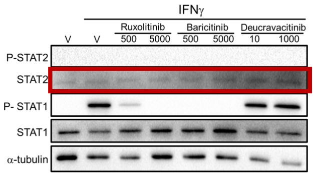

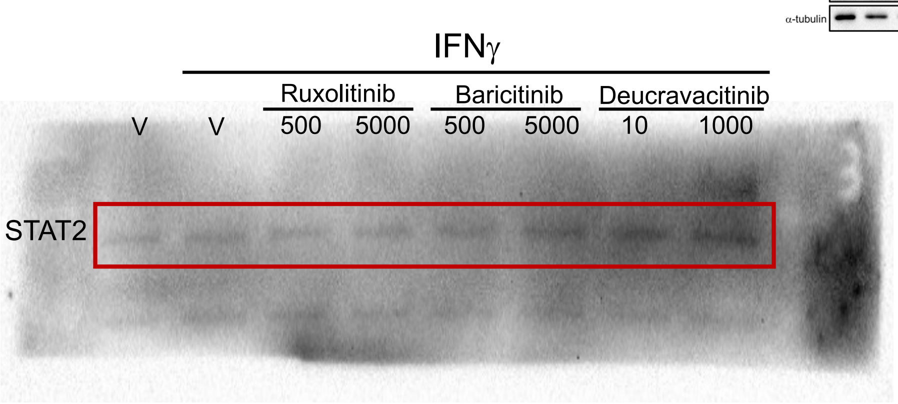

Full unedited blot for **Figure 2C**

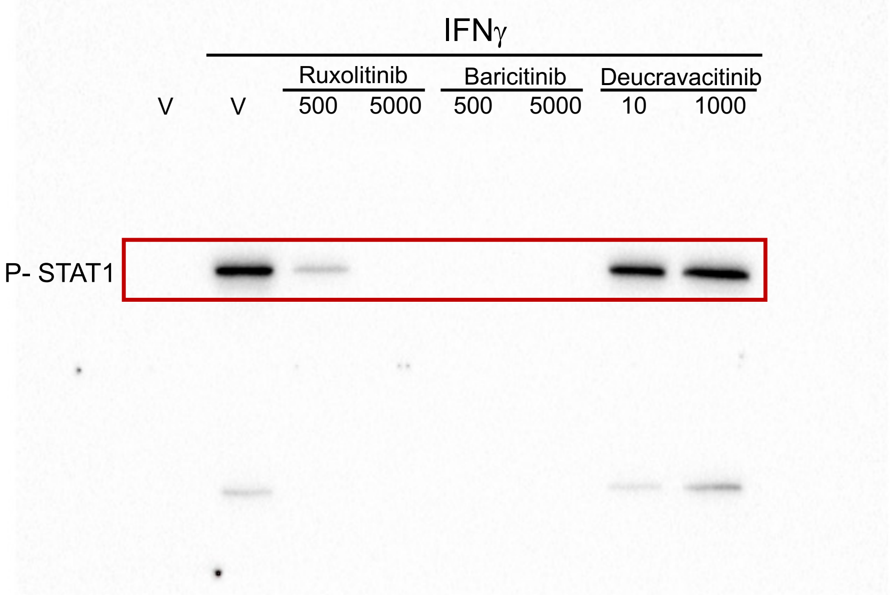

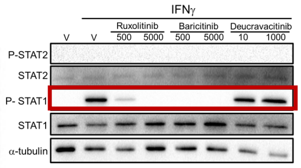

Full unedited blot for **Figure 2C**

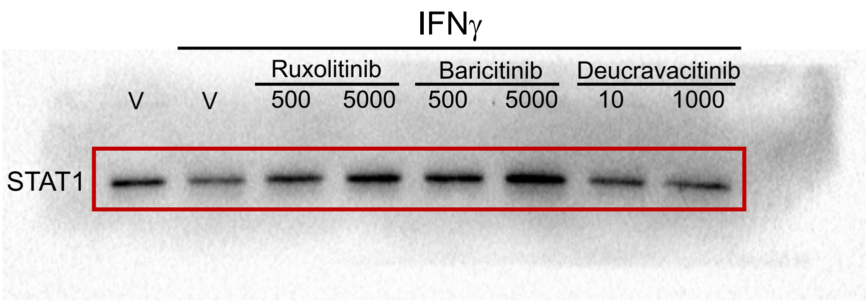

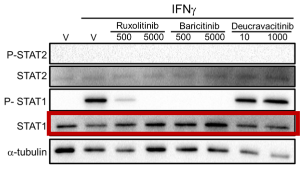

Full unedited blot for **Figure 2C**

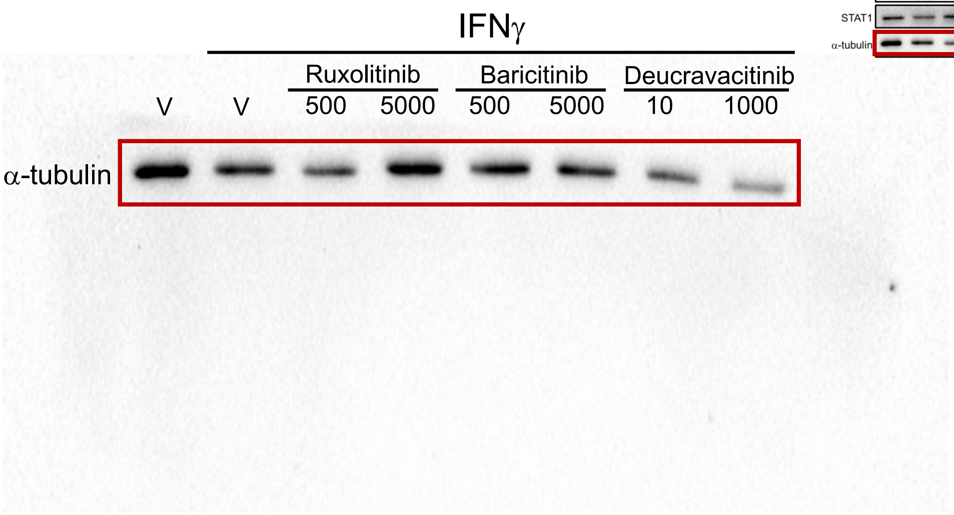

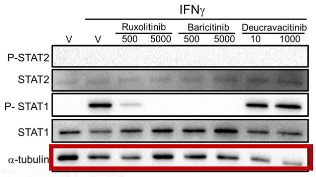

Figure 3D

Full unedited blot for **Figure 3D**

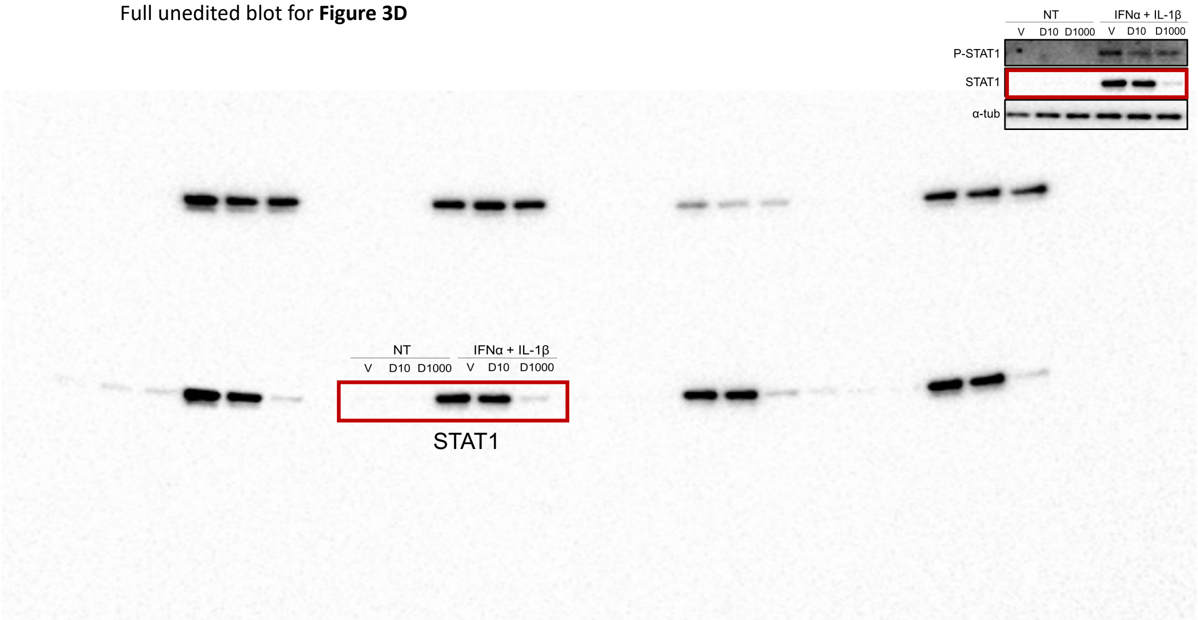

Full unedited blot for **Figure 3D**

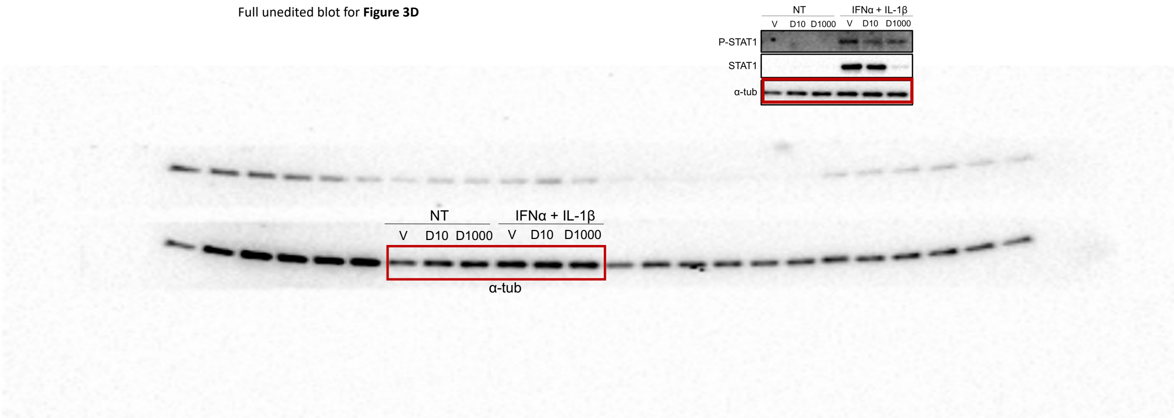

Figure 3H

Full unedited photo for **Figure 3H**

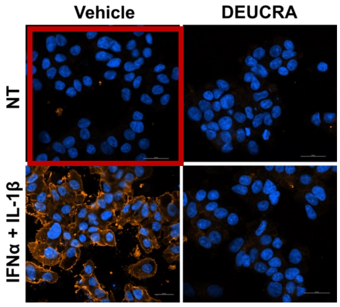

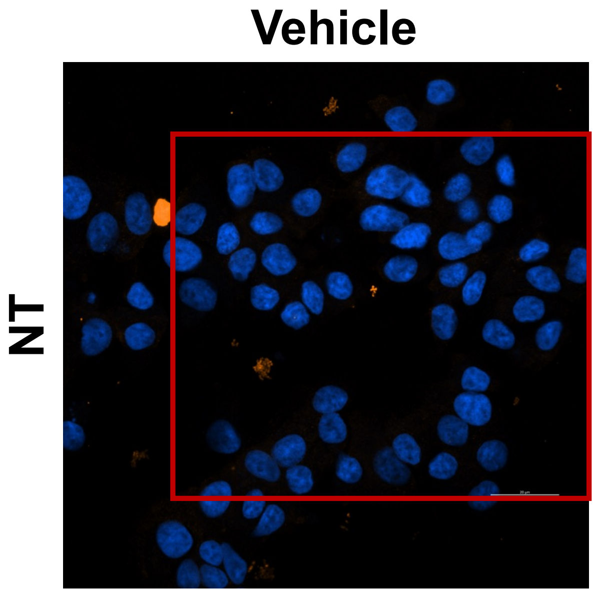

Full unedited photo for **Figure 3H**

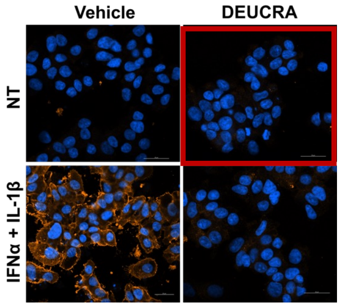

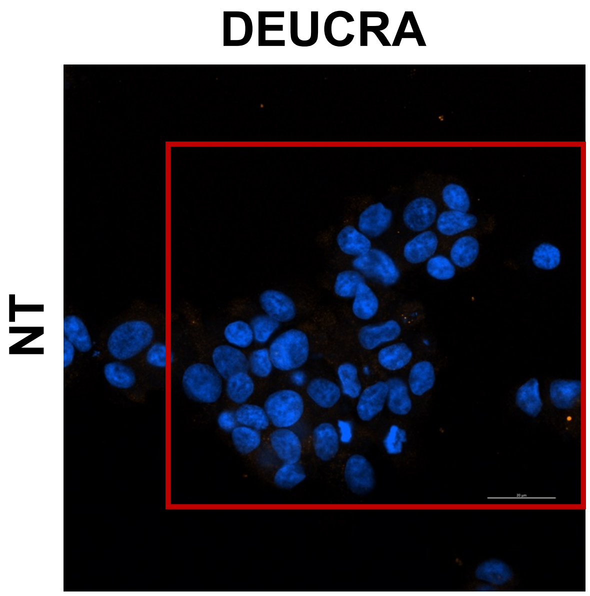

Full unedited photo for **Figure 3H**

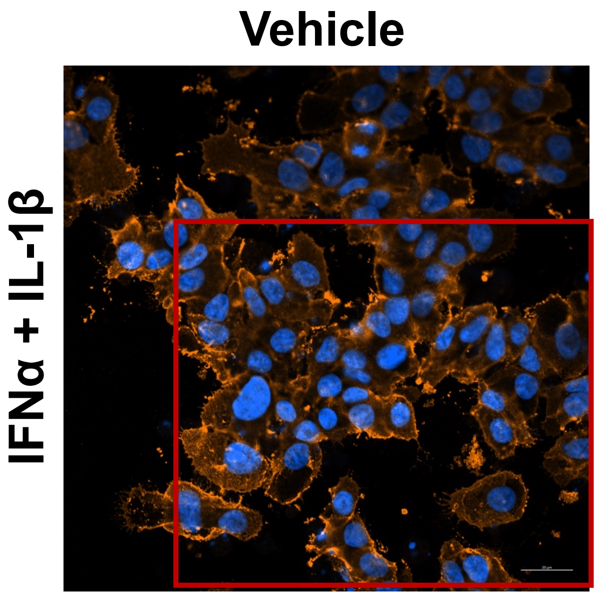

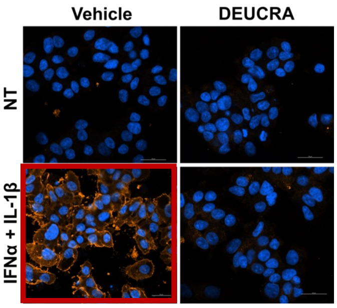

Full unedited photo for **Figure 3H**

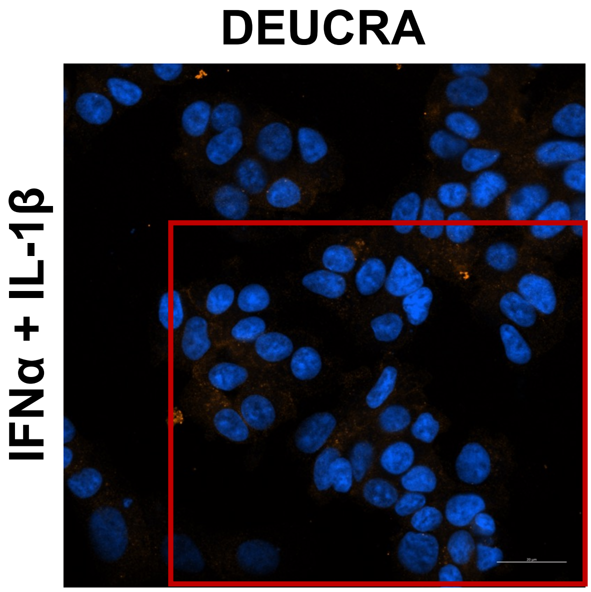

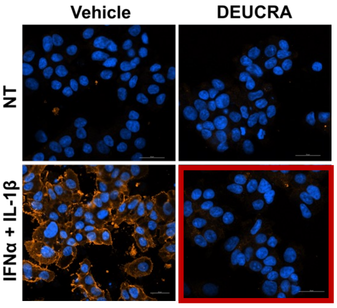

Figure 3N

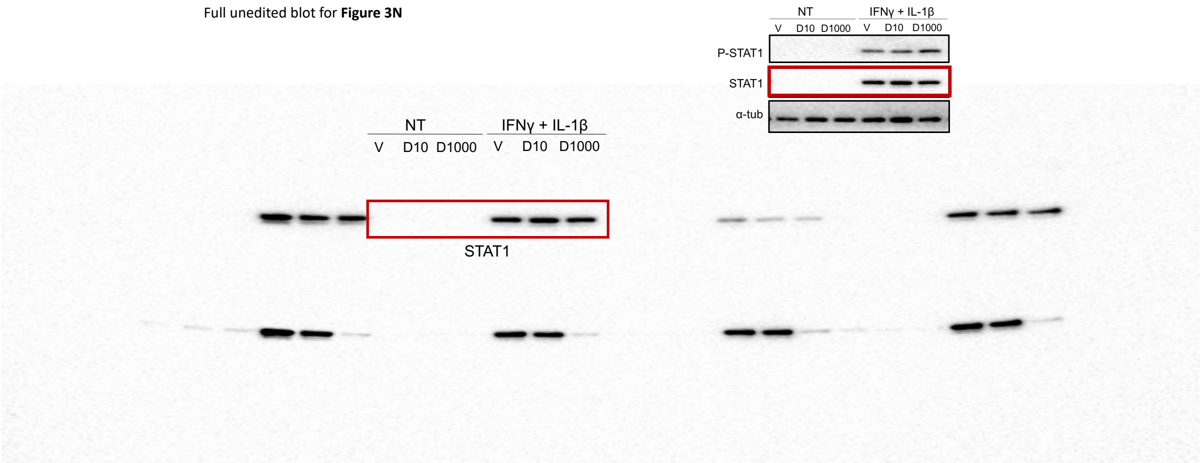
Full unedited blot for **Figure 3N**
NT
IFNγ + IL-1β
V
D10
D1000
V
D10
D1000
P-STAT1
STAT1
α-tub
STAT1

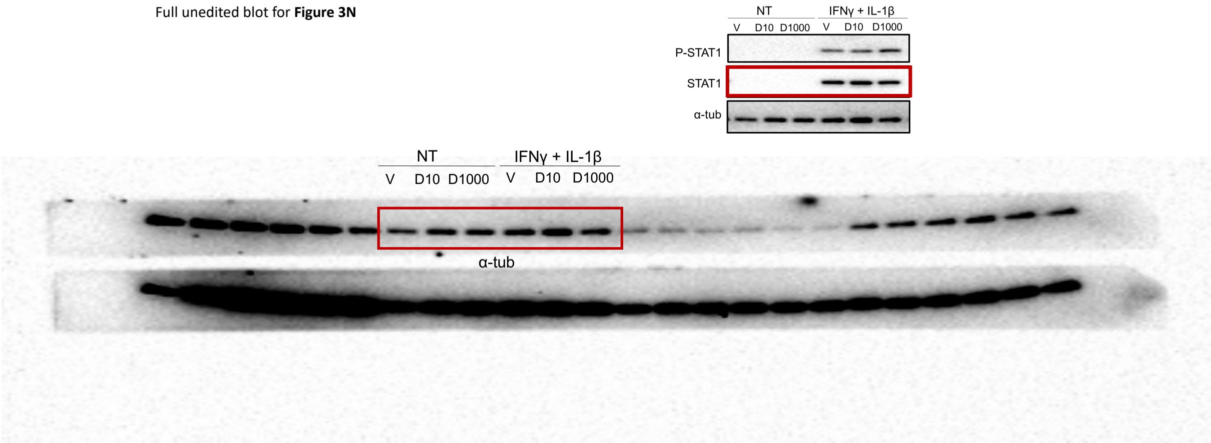
Full unedited blot for **Figure 3N**
NT
IFNγ + IL-1β
V
D10
D1000
V
D10
D1000
P-STAT1
STAT1
α-tub
α-tub

Figure 4C

Full unedited blot for **Figure 4C**

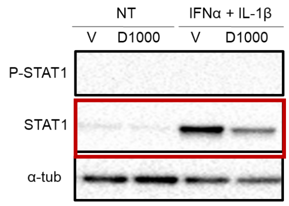

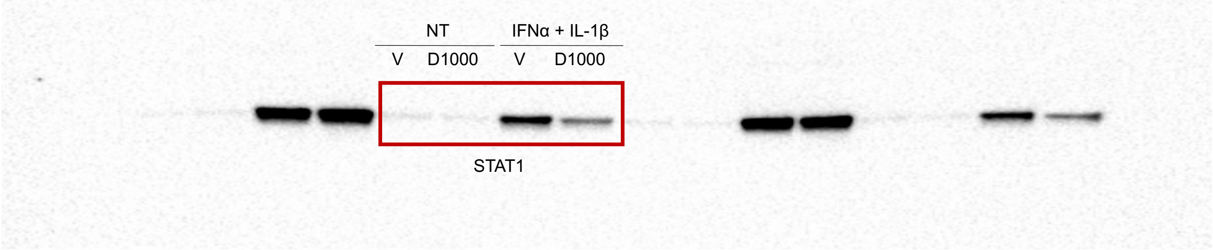

Full unedited blot for **Figure 4C**

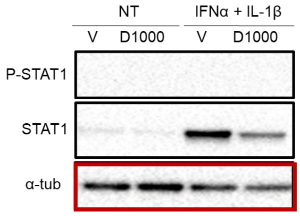

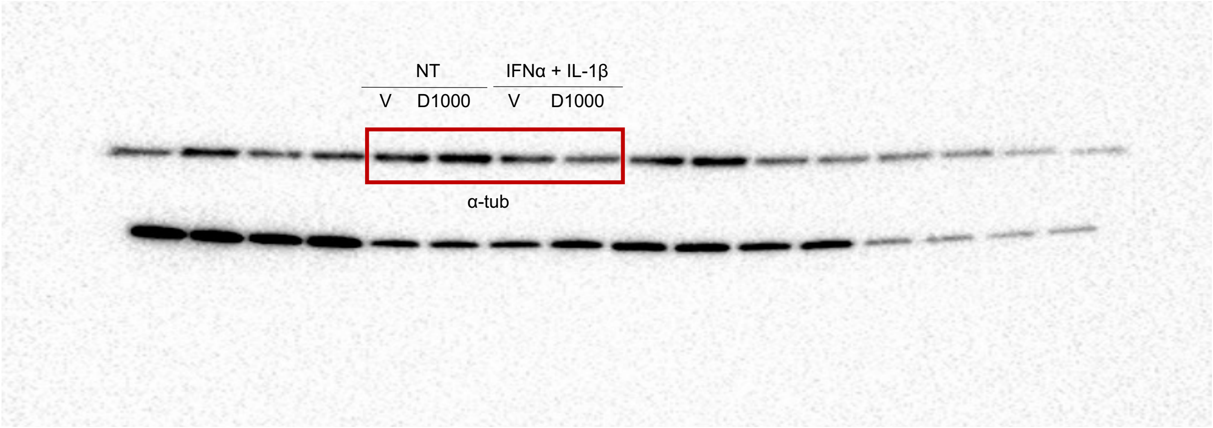

Figure 4J

Full unedited blot for **Figure 4J**

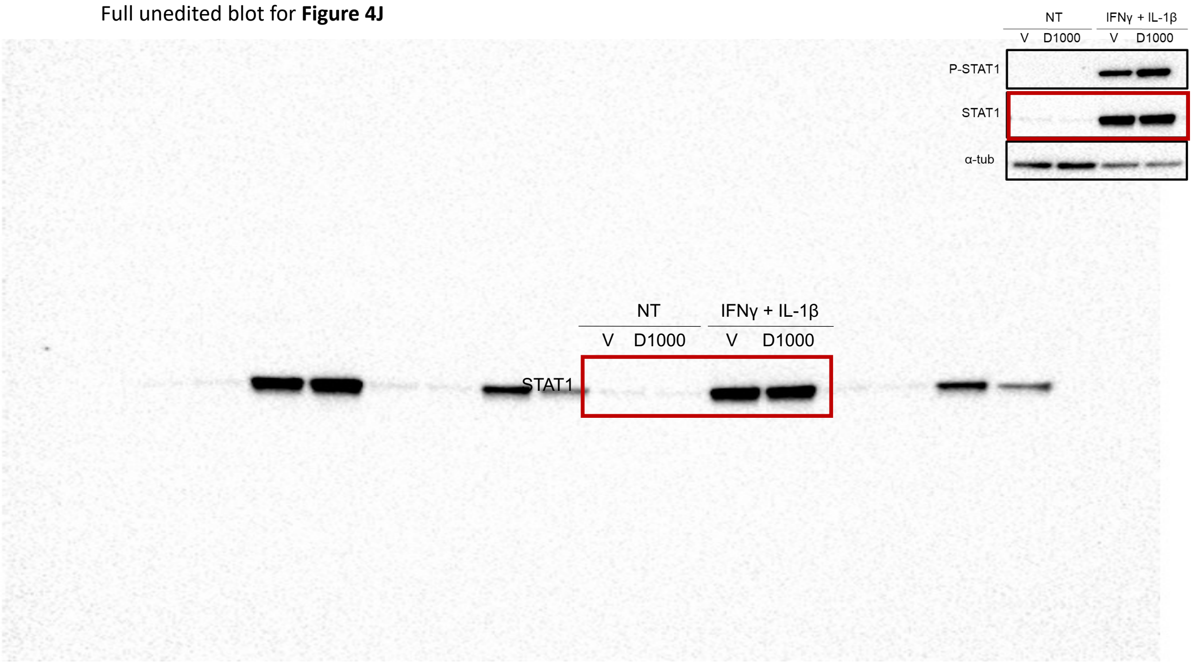

Full unedited blot for **Figure 4J**

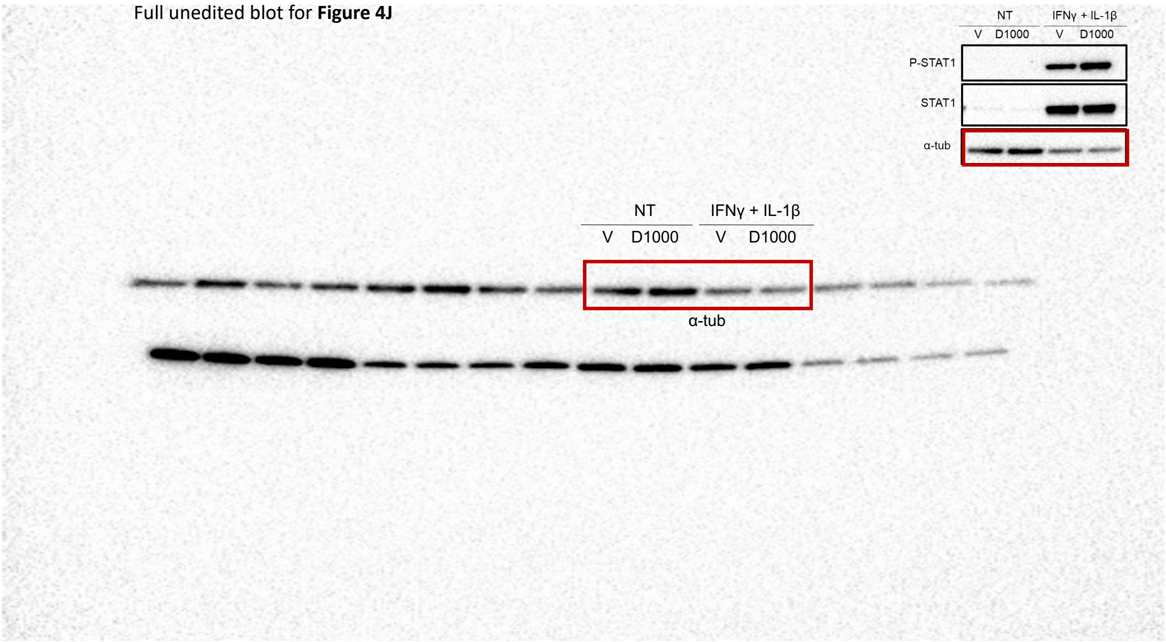

Supplement: Supplementary file 1 [file DataSheet_1.pdf]
